# Folate-Targeted Curcumin-Loaded Niosomes for Site-Specific Delivery in Breast Cancer Treatment: In Silico and In Vitro Study

**DOI:** 10.3390/molecules27144634

**Published:** 2022-07-20

**Authors:** Banafsheh Honarvari, Sara Karimifard, Niyayesh Akhtari, Mehrnoush Mehrarya, Zahra Salehi Moghaddam, Mohammad Javed Ansari, Abduladheem Turki Jalil, Adrián Matencio, Francesco Trotta, Faten Eshrati Yeganeh, Bahareh Farasati Far, Mandana Kazem Arki, Mohammad Reza Naimi-Jamal, Hassan Noorbazargan, Zahra Asghari Lalami, Mohsen Chiani

**Affiliations:** 1 Department of Physics, Faculty of Physics and Chemistry, Alzahra University, Tehran 1993893973, Iran; banafshehhonarvari@gmail.com; 2 Central Tehran Branch, Stem Cells Research Center, Tissue Engineering and Regenerative Medicine Institute, Islamic Azad University, Tehran 1568937813, Iran; karimifard.sara@gmail.com; 3 Department of Biology, Parand Branch, Islamic Azad University, Parand 3761396361, Iran; niyayeshakhtari@gmail.com; 4 Protein Research Center, Shahid Beheshti University, Tehran 1983969411, Iran; mehrnoush1995@yahoo.com; 5 Department of Microbial Biotechnology, School of Biology, College of Science, University of Tehran, Tehran 141556455, Iran; salehi.moghaddam@ut.ac.ir; 6 Department of Pharmaceutics, College of Pharmacy, Prince Sattam Bin Abdulaziz University, Al-Kharj 16278, Saudi Arabia; javedpharma@yahoo.com; 7 Medical Laboratories Techniques Department, Al-Mustaqbal University College, Babylon, Hilla 51001, Iraq; abedalazeem799@gmail.com; 8 Department of Chemistry, University of Turin, Via Pietro Giuria 7, 10125 Torino, Italy; adrian.matencioduran@unito.it (A.M.); francesco.trotta@unito.it (F.T.); 9 Department of Chemistry, Science and Research Branch, Islamic Azad University, Tehran 14515775, Iran; ffyeganeh@gmail.com; 10 Research Laboratory of Green Organic Synthesis and Polymers, Department of Chemistry, Iran University of Science and Technology, Tehran 1684611367, Iran; bahar.ferasati@gmail.com (B.F.F.); naimi@iust.ac.ir (M.R.N.-J.); 11 Gastroenterology and Liver Diseases Research Center, Research Institute for Gastroenterology and Liver Diseases, Shahid Beheshti University of Medical Sciences, Tehran 19835178, Iran; mandana.arki@gmail.com; 12 Department of Biotechnology, School of Advanced Technologies in Medicine, Shahid Beheshti University of Medical Sciences, Tehran 1985717443, Iran; h.noorbazargan@sbmu.ac.ir; 13 New Technology Research Group, Department of Nano Biotechnology, Pasteur Institute of Iran, Tehran 1316943551, Iran

**Keywords:** curcumin, breast cancer, endocytosis, folic acid, PEG, niosome, in silico studies, ADME prediction, molecular docking

## Abstract

As the most common cancer in women, efforts have been made to develop novel nanomedicine-based therapeutics for breast cancer. In the present study, the in silico curcumin (Cur) properties were investigated, and we found some important drawbacks of Cur. To enhance cancer therapeutics of Cur, three different nonionic surfactants (span 20, 60, and 80) were used to prepare various Cur-loaded niosomes (Nio-Cur). Then, fabricated Nio-Cur were decorated with folic acid (FA) and polyethylene glycol (PEG) for breast cancer suppression. For PEG-FA@Nio-Cur, the gene expression levels of Bax and p53 were higher compared to free drug and Nio-Cur. With PEG-FA-decorated Nio-Cur, levels of Bcl2 were lower than the free drug and Nio-Cur. When MCF7 and 4T1 cell uptake tests of PEG-FA@Nio-Cur and Nio-Cur were investigated, the results showed that the PEG-FA-modified niosomes exhibited the most preponderant endocytosis. In vitro experiments demonstrate that PEG-FA@Nio-Cur is a promising strategy for the delivery of Cur in breast cancer therapy. Breast cancer cells absorbed the prepared nanoformulations and exhibited sustained drug release characteristics.

## 1. Introduction

Breast cancer is the most common malignant tumor in women (30% of all cancer cases) and is responsible for the second-most cancer-related deaths (15% of all cancer deaths). Based on new estimates, the incidence rate of breast cancer will increase annually by almost 0.5% due to reduced fertility and overweight [1]. The 5-year survival rate of breast cancer is 90%, and notably, the survival rate is lower in black patients than white. Breast cancer mortality from 1989 to 2018 has demonstrated a decrease of 41%. However, the reduction in mortality has slowed for breast cancer in recent years. Breast cancer is more common in women aged 20 to 59 years [2,3,4]. Regardless of epidemiology, breast cancer is heterogeneous, and tumor cells demonstrate high proliferation and migration rates. Due to aggressive behavior and switching among various molecular pathways to increase progression, breast cancer cells can achieve resistance to therapies, especially chemotherapy [5]. Therefore, efforts have been made to find new antitumor compounds for breast cancer, and phytochemicals are of interest [6].

Curcumin (Cur) (1,7-bis (4-hydroxy-3-methoxyphenyl)-1,6-heptadiene-3,5-dione) is a bioactive compound isolated from the root and rhizome of Curcuma longa and has a yellow color with multiple applications in food and pharmaceutical industries. It has two enol and keto forms; the enol form is observed in alkaline solutions, while the ketol form occurs in neutral and acidic pH [7]. Cur is well-known for its health-promoting impacts such as antidiabetic, antimicrobial, antioxidant, anti-inflammatory, and anticancer activities [8]. Cur has the capacity to induce apoptosis in cancer cells and reduces metastasis via EMT inhibition. Furthermore, Cur supplementation promotes sensitivity to cancer chemotherapy [9]. However, the health benefits of Cur are limited due to poor bioavailability, so that upon oral administration, a low amount of this valuable compound is absorbed by the intestine and excretion occurs for the rest. The rapid metabolism of Cur in the liver and plasma also decreases its bioavailability [10]. Hence, nanoscale delivery systems have been designed for Cur in cancer therapy, especially breast cancer [11].

Among nanostructures, niosomes have demonstrated a promising profile in cancer drug delivery. Niosomes possess an aqueous core coated with the lipid bilayer. The niosomes are comprised of nonionic surfactants and cholesterol (as the lipid), and they show high stability and biocompatibility that are important for drug delivery. Both hydrophobic and hydrophilic drugs can be delivered by niosomes [12,13]. The safety profile of niosomes is higher than other nanoarchitectures due to the biocompatible nature of their elements [14,15]. Furthermore, surfactants in niosomal formulations undergo hydrolysis in acidic pH, rendering a pH-sensitive feature that leads to burst release in acidic media [16,17]. The PEGylation of niosomes improves their biocompatibility and makes it possible to provide surface modification [18]. Moreover, PEGylation is important in prolonging nanostructured circulation time and preventing burst release. On the other hand, folate receptors show upregulation in breast cancer cells, while their expression level is low in normal cells [19]. Hence, conjugating folic acid (FA) to nanostructures results in the selective targeting of breast cancer cells overexpressing folate receptors [20].

In this study, we take all the above cited together to create a novel niosome-based formulation of Cur, which could present higher circulation time and target delivery. These points could decrease the total Cur dosage optimizing the delivery and future treatment. To check this hypothesis, we developed highly biocompatible Cur-loaded niosomes (Nio-Cur) based on spans 20, 60, and 80. The effects of surfactant type on particle size, polydispersity index (PDI), and efficiency of drug encapsulation (EE%) were investigated. These nanostructures improved the bioavailability of Cur and increased intracellular accumulation in cancer cells via the endocytic pathway. The synthesis of Cur-loaded PEG@FA niosomes with span 80 was carried out by the thin-film hydration and conjugation chemistry method. The advantage of using PEG is to conjugate FA on the surface of niosomes. Subsequently, we compared the stability and drug release of Nio-Cur and PEG-FA@Nio-Cur. One of our work’s advantages was reducing the release of Cur from PEG-FA@Nio-Cur compared with the Nio-Cur. Cell cytotoxicity and apoptosis were assessed. Gene expression was studied using a real-time polymerase chain reaction.

## 2. Material and Methods

### 2.1. In Silico Study

#### 2.1.1. Recognition of Target’s Properties

The amino acid sequence of the Bcl2, Bax, and p53 protein was received from UniProtKB (Uniprot ID: P10415, Q07812, P04637, respectively). The crystal structures of proteins were selected from the Protein Data Bank (https://www.rcsb.org/). PDB proteins were selected based on coverage, resolution, and R-value. protein physicochemical properties were investigated by inputting the FASTA file to the ProtParam server (https://web.expasy.org/protparam/) [21].

#### 2.1.2. Validation of Bcl2, Bax, and p53

The protein structures were validated by the SAVES v6.0 server (https://saves.mbi.ucla.edu/) and ProSA (Protein Structure Analysis) (https://prosa.services.came.sbg.ac.at/). The ProSA-web server indicated a z-score using the PDB file or PDB code to recognize the experimental and theoretical protein structures [22,23]. Six different programs were evaluated in the SAVES server. We used Ramachandran plot [24], ERRAT [25], and Verify3D [26,27] for validation after submitting the PDB file.

#### 2.1.3. ADME Prediction

ADME (absorption, distribution, metabolism, and excretion) pharmacokinetic knowledge is an important part of in silico study from the early stages to the final clinical evaluation [28,29]. Curcumin’s bioavailability and membrane permeability are usually linked to basic molecular characteristics such as Log P (partition coefficient), molecular weight (MW), or the quantity of hydrogen bond acceptors and donors in molecules [30]. These molecular characteristics were examined in detail by Lipinski’s “rule of five” [31]. Curcumin ADME was calculated by submitting a SMILE format of curcumin to ADMET Lab Webserver (http://admet.scbdd.com/calcpre/index/) and Swiss ADME (http://www.swissadme.ch/). Curcumin SMILE format was obtained from the PubChem database ((https://pubchem.ncbi.nlm.nih.gov/). Furthermore, ADME properties, including Caco-2 permeability, bioavailability 30% (F30), plasma protein binding (PPB), blood–brain barrier (BBB), Cyp450 1A2 inhibitor, Cyp450 1A2 substrate, half-life (T1/2), and clearance (CL), were calculated in the current research [32].

#### 2.1.4. Ligand Preparation and Molecular Docking

Molecular docking mimics the biological systems and predicts the interaction of curcumin with Bax, Bcl2, and p53 genes. Curcumin (SDF format files) was taken from the pubchem server (https://pubchem.ncbi.nlm.nih.gov/). The native PDB proteins’ structures were unsuitable for molecular docking due to unnecessary molecules [33]. Thus, cocrystallized ligands and solvents were removed by UCSF Chimera v1.8.1. Moreover, polar hydrogens were added by a bash script in the Linux system [34]. PDB structures of proteins were opened in the PyRx v.0.8 virtual screening tool. Ligands’ energy was minimized and converted to PDBQT format in AutoDock tools v1.5.6 [35]. AutoDock Vina (1.1.2) performed self-docking simulations [36]. Afterward, the maximum size of the grid box, approximately (x = 35.09, y = 44.44, z = 52.15 Å), was chosen. Conformers of curcumin underwent screening according to binding affinity and RMSD. Binding affinity and RMSD are significant factors for investigating interaction among curcumin and targets. Moreover, 2D interactions of curcumin with proteins were visualized by Discovery Studio3.1 software [37,38].

#### 2.1.5. Molecular Dynamics Simulation

The molecular dynamics (MD) simulation was performed to investigate the stability and binding affinity between curcumin and modeled proteins (p53, Bax, and Bcl2). MD simulations were performed using the GROMACS (v2021.2) [39] software package. The initial structure for MD simulation was obtained from the docking process. In this research, we used the CHARMM 27 force field. To gain force field parameters of curcumin, we used SwissParam [40]. After adding the TIP3P water model as a solvent to the simulation box, brief energy minimization was carried out. Then, the systems were equilibrated under NVT and NPT ensembles, making the system’s temperature and pressure constant at 300 K and 1 bar, respectively. A v-rescale thermostat [41] and Parrinello–Rahman barostat [42] were used for equilibration. Following equilibration, a 40 ns MD run was carried out in which the time step was set to 2 fs. The leap-frog algorithm was used to integrate the equation of motion. During MD simulation, periodic boundary conditions (PBCs) were applied in three dimensions. The electrostatic interactions were calculated using particle mesh Ewald (PME) [43] with a coulomb cutoff of one nanometer. Additionally, van der Waals interactions cut off at one nm. Additionally, all bonds were constrained by employing a linear constraint solver (LINCS) algorithm [44]. In this research, we collected trajectories every ten ps.

### 2.2. In Vitro Study and Niosome Synthesis and Characterization

#### 2.2.1. Materials

The following products were bought from Millipore Sigma (Burlington, MA, USA) and utilized without any extra-purification: curcumin (Cur), Span^®^ 20 (Sorbitan monolaurate), Span^®^ 60 (sorbitan monostearate), Span^®^ 80 (Sorbitan monooleate), and cholesterol. The following materials were purchased from Merck Chemical Co. (Darmstadt, Germany): chloroform, methanol, FA-PEG-derivatized mPEG2000-DSPE (FA-PEG2000-DSPE), dimethyl sulfoxide (DMSO), dialysis membrane (MWCO 12,000 Da), sodium dodecyl sulfate (SDS), DCP (dicetylphosphate), phosphate-buffered saline (PBS), and paraformaldehyde. The Pasteur Institute Cell Bank (Tehran, Iran) provided the MCF10A (NCBI Code, C106), MCF7 (NCBI Code, C135), and 4T1 (C604) cell lines. Formaldehyde, trypsin-EDTA, Trypan blue, fetal bovine serum (FBS), RPMI-1640 medium, phosphate-buffered saline, 3-(4,5-dimethylthiazol-2-yl)-2,5-diphenyltetrazolium bromide (MTT), and penicillin/streptomycin (PS) 100 X were purchased from Gibco, ThermoFisher Scientific RPMI-1640 (Waltham, MA, USA). Affymetrix Biosciences and ThermoFisher Scientific provided the 1X binding buffer and annexin V-FITC flow cytometry kit. Transgene Biotech Ltd. (Beijing, China) supplied the RNA Extraction and cDNA Synthesis kits (Cat Nos. ER101-01 and AE301-02). The DCFDA/H2DCFDA Assay Kit for Cellular Reactive Oxygen Species (ROS) was purchased from Teb Pazhouhan Razi (TPR), Tehran, Iran, Code No. TPR-ROS-96T). Calbiochem (San Diego, CA, USA) supplied the propidium iodide DAPI and TritonTM X-100 (4,6-diamidino-2-phenylindole dihydrochloride).

#### 2.2.2. Synthesis of Niosomal Formulations

Niosomes loaded with curcumin (Nio-Cur) were prepared using the thin-film hydration method [45,46]. Briefly, curcumin, cholesterol, surfactants (Span^®^ 20, Span^®^ 60, and Span^®^ 80), and 9 mL of chloroform/methanol (2:1; *v*/*v*) were used to dissolve DCP. A 50 mL round bottom flask was used to collect the solution. To dry the organic solvent, we used a Heidolph Rotary Evaporator (Schwabach, Germany) set at 60 °C and 150 rpm to evaporate it under a vacuum for 30 min. The thin film was rehydrated using PBS (1X, 10 mL, pH 7.4). The reactants were subsequently dried at 60 °C and 150 rpm for 30 min to obtain dried specimens. A Hielscher Ultrasonics UP50H small laboratory homogenizer (Teltow, Germany) was used to homogenize the Nio-Cur specimens to ensure that they had an appropriate size distribution, i.e., homogeneous particle dispersion. The specimens were kept at 4 °C until the characterization of the different niosomal formulations listed in Table S1 (Supplementary Materials).

#### 2.2.3. Surface Functionalization of the Optimized Niosomal Formulation

Niosomes coated with PEG-folic acid (PEG-FA@Nio-Cur) were synthesized by dissolving Span^®^ 80, cholesterol, DCP, and FA-PEG2000-DSPE in 9 mL of chloroform/methanol (2:1; *v*/*v*) containing 10 mg dissolved curcumin.

#### 2.2.4. Physical Characterization of Niosomal Formulations

Benchtop dynamic light scattering/electrophoretic light scattering systems were used to measure particle size, polydispersity index (PDI), and zeta potential in niosomal formulations at 25 °C (Zetasizer Nano S90, Malvern Panalytical Ltd., Malvern, UK). A scanning electron microscope (SEM) and transmission electron microscope (TEM) were used to examine the specimens’ morphology (microscope SSX-500, Shimadzu, Kyoto, Japan). A diluted sample (1:100) was placed on the SEM holder (attached to aluminum stubs with carbon tape) and coated with a layer of 100 Å gold for 3 min under argon at a pressure of 0.2 atm [47,48]. For TEM imaging, a drop of niosomal formulations was placed on a carbon-coated copper grid and stained with a 1% phosphotungstic acid. The thin film of stained niosomes was imaged with a TEM (Zeiss EM900 Transmission Electron Microscope, Carl Zeiss AG, Jena, Germany) at 100 kV.

#### 2.2.5. Entrapment Efficacy

An Amicon Ultra-15-membrane was used to ultrafilter the niosomal formulations (Eppendorf^®^ 580R centrifuge, Hamburg, Germany) or centrifugal filter—30Kda molecular weight cutoff—at 4000 rpm for 20 min, at 4 °C. To estimate the effectiveness of entrapment, the non-entrapped Cur was separated from the entrapped Cur (EE percent). The UV-Visible light spectrophotometer was used to measure the free “drug” concentration at 420 nm (UV-1700 PharmaSpec, Shimadzu, Kyoto, Japan). To compute EE percentage, the following formula was used [49]:

EE%=[(A−B)/A]×100

where *A* is the initial Cur concentration for the niosomal preparation and *B* is the concentration of non-entrapped Cur after centrifugation.

#### 2.2.6. In Vitro Drug Release

Dialysis bags were used to test drug release by placing 2 mL of each sample in them (MWCO 12 kDa). To immerse it, 50 mL of PBS-SDS (0.5% *w*/*v*) release media was used. A magnetic stirrer was used to agitate the mixture at 50 rpm under various pH settings. (7.4 and 5.4 physiological (~7.4) and pathological cancerous (~5.4)) at different temperatures (25 °C and 37 °C) for 72 h. A particular amount of release medium was withdrawn and replaced with the same amount of fresh PBS-SDS at a predetermined time interval (1, 2, 4, 8, 24, 48, and 72 h) [50]. An ultraviolet-light spectrophotometer was used to assess the amount of drug delivered at predetermined intervals. An equal concentration of drugs inside and outside the dialysis bag was used as a control in this test.

The Higuchi model, the Korsmeyer–Peppas model (log cumulative % drug release vs. log time), the first-order model (cumulative % drug remaining in samples vs. time), and the zero-order model (cumulative % drug remaining vs. time) were all used to assess the release kinetics of Cur from samples (cumulative % drug release vs. time) [51,52,53,54]. To calculate the linear curve, the correlation coefficient (r) values obtained by regressing the plots acquired from the models above were employed. The zero-order model represents a system in which the drug release rate is unaffected by concentration since it relies on drug dissolution. The rate at which a drug is released is proportional to the concentration of that drug, as described by a first-order rate equation [51]. Models such as those proposed by Higuchi and Korsmeyer–Peppas show a direct correlation between the amount of medication released from a matrix system and its square root [55,56]. The first release of 60% was sufficient for finding the best model for drug release [57].

#### 2.2.7. Stability

The Nio-Cur and PEG-FA@Nio-Cur samples were stored at 25 ± 1 °C or 4 ± 1 °C, at 60 ± 5% relative humidity for one month. Particle size, PDI, and % of active ingredients were tested to determine the stability of the formulations after they had been stored.

#### 2.2.8. Culture of MCF7 and 4T1 Cell Lines

Mammary cancer cell lines MCF7 and 4T1 from humans and mice were grown at 37 °C in a mixture of ambient acid and 5% CO_2_. The culture media was RPMI-1640 fresh medium with 10% FBS and 1% penicillin/streptomycin (complete growth medium). After the cells achieved 85% to 95% confluence, the media was aspirated. Trypsin-EDTA, 0.25% (*w*/*v*), was used to detach the cell monolayer. The trypan-blue-labeled cells were resuspended in full growth media and counted using a hemocytometer after removing them.

#### 2.2.9. Cell Proliferation

Different concentrations of Cur, Nio-Cur, or PEG-FA@Nio-Cur (800, 400, 200, 100, 50, 25, 12.5, and 6.25 μg/mL) were applied to cultivated MCF7 and 4T1 cells and incubated for 48 and 72 h, respectively, before the results were collected. For control, different dilutions of empty niosomes (Nio) and different concentrations of Cur, Nio-Cur, or PEG-FA@Nio-Cur (800, 400, 200, 100, 50, 25, 12.5, and 6.25 μg/mL) were added to a nontumorigenic epithelial cell line (MCF10A) and incubated for 48 h and 72 h. Different concentrations of Cur, Nio-Cur, or PEG-FA@Nio-Cur were applied to cultivated MCF7 and 4T1 cells and incubated for 48 and 72 h, respectively, before the results were collected. Different dilutions of empty niosomes were used as a control (Nio). To colorimetrically assess the oxidoreductase enzyme activity in cells, the formazan was dissolved in 100 L of DMSO. The following formula was used to calculate the results:

Percentage cell viability (%) = Optical Density 570–630 treatment/Optical Density 570–630 control × 100%

#### 2.2.10. Apoptosis

To determine the proportion of apoptotic MCF7 and 4T1 cells, a flow cytometry technique was used. Nio, Cur, Nio-Cur, or PEG-FA@Nio-Cur were all used to treat the cells for 48 h. Fluorescence labeling for flow cytometry was performed using the Apoptosis and Necrosis Quantitation Kit to identify necrotic and apoptotic cells. They were resuspended in 1X binding buffer (5 × 105 cells/well) and washed twice with PBS. An annexin V-FITC (green fluorescence) and propidium iodide (red fluorescence) staining procedure were followed before the cells were examined using a benchtop flow cytometer (FACSCalibur, BD Biosciences, Franklin Lakes, NJ, USA).

#### 2.2.11. Cell Cycle

PBS was used to wash the cells once; then, 50 L of PBS was added to the cells and vortexed once the incubation periods were complete. In the following phase, a fixative of 1 mL cold 70% ethanol was added to the samples, and the vortexing was repeated. To avoid clumping, the cells were shaken during the fixation period. Once centrifuged and rinsed with PBS, the cells were refrigerated in the fridge for two h before centrifugation and washing. For this experiment, cells were prewashed with PBS and then given one milliliter of the PI master mix solution, containing 40 μL PI, 10 μL of RNase (0.5 μg/mL), 1 mL of PBS, and 0.1% (*v*/*v*) Triton X-100. Flow cytometry was used to analyze the cells after being cultured for 30 min at room temperature. FlowJo software was used to examine the data.

#### 2.2.12. Reactive Oxygen Species

The H2DCFDA kit assessed the ROS produced by samples (2′,7′-dichlorodihydrofluorescein diacetate). The fluorescence of 2′,7′-dichlorodihydrofluorescein diacetate acetyl esters (which was generated via the DCFDA reduction) can be assigned to cellular oxidation. It could be an indicator of oxidative stress. The most susceptible MCF7 and 4T1 cell lines were treated with IC50 concentrations of samples for 48 h. The cells were washed in buffer saline before 30 min of incubation at 37 °C with 80 mM H2DCFDA. A microplate reader was used to quantify the fluorescence intensity at 530 nm.

#### 2.2.13. Quantitative Real-Time Polymerase Chain Reaction (qRT-PCR)

The two types of cancer cells (1 × 108 cells/well) were treated with Nio, Cur, Nio-Cur, or PEG-FA@Nio-Cur for 48 h. Afterward, the cells were rinsed with PBS, and 50 L of PBS was added, followed by a vortexing step. As a fixative, 1 mL of cold 70% ethanol was added to the samples and vortexed once more. The clumps were prevented by shaking the cells while they were fixed. After 2 h in the fridge, the cells were centrifuged and washed with PBS once each. An amount of 1 mL of the PI master mix solution was introduced to the cells, along with one milliliter of PBS containing 0.1% (*v*/*v*) Triton X-100, and the PBS was gently withdrawn. After a 30-min incubation period at room temperature, the cells were analyzed using flow cytometry. A software program called FlowJo was used to examine the data (30 µL).

The extracted RNA was used to generate complementary DNA (cDNA) by adding 10 μL of reaction buffer (2×), 5 g of the RNA, and 2 μL of Enzyme-Mix to 20 L of DEPC-treated water in RNase-free tubes. It was incubated for 10 min at 25 °C and then for 60 min at 47 °C before being analyzed. Afterward, the combination was held on ice until ready to use by heating it to 85 °C for 5 min.

The National Center for Biotechnology Database was used to construct the primers for Bax, Bcl2, p53, and β-actin. For each of these genes, the forward and reverse primer sequences may be found in Table S2. A 7500 Real-time PCR System was used to run the qRT-PCR utilizing sense and antisense primers (Applied Biosystems, Carlsbard, CA, USA). The housekeeping gene was β-actin. The fold changes in comparison to the control group were determined using the 2^–ΔΔCT^ technique. Triple-blind studies were carried out for each experiment. Additionally, qRT-PCR analysis with a new housekeeping gene (PBGD) was performed.

#### 2.2.14. Cellular Uptake of Formulations

Confocal microscopy was used to study the cellular uptake of different formulations in MCF7 and 4T1 cells. MCF7 and 4T1 (1 × 105 cells per well) were grown on coverslips placed in 24-well plates for 24 h. On attaining 80% confluency, the cells were treated with IC50 concentration of Cur, Nio-Cur, or PEG-FA@Nio-Cur. After 2 h of incubation, the cells were rinsed in PBS and fixed with PFA. A confocal laser scanning microscope in the FITC channel was used to examine curcumin intracellular fluorescence in the nuclei, which were then mounted in DPX and stained with propidium iodide (488 nm).

#### 2.2.15. DAPI Staining

The nuclear morphology of apoptotic cells was investigated using DAPI staining. MCF7 and 4T1 cells were plated onto a 24-well plate and exposed to the IC50 concentration of formulations for 48 h. The MCF7 and 4T1 cells were then fixed with 4% paraformaldehyde. The MCF7 and 4T1 cells were then washed in PBS and permeated for 10 min with 0.1% Triton X-100. The cells were stained with DAPI for 10 min after being washed in PBS. Ultimately, the cell morphologies were studied using an Olympus IX81 inverted fluorescence microscope with a DP72 digital camera (Olympus, Hamburg, Germany) [58].

### 2.3. Statistical Analysis

After a 2-h incubation period, the cells were rinsed in PBS and fixed in PFA before being placed back in the dish. A confocal laser scanning microscope in the FITC channel was used to examine curcumin intracellular fluorescence in the nuclei, which were then mounted in DPX and stained with propidium iodide (488 nm). For all analyses, statistical significance was pre-set at α = 0.05.

## 3. Results

### 3.1. In Silico Study

#### 3.1.1. Recognition of Target Properties

Among 26 (3D) structures for the Bax protein, we selected the 6EB6 PDB (Protein Data Bank) structure which has the lowest resolution (2.02 Å), the precise position of residues (1–92), and covers the length of the protein. Moreover, 6GL8 PDB structures (for Bcl2) with 1.40 Å resolution were chosen among 27 NMR and X-ray structures. Furthermore, the third gene, p53 (6SL6 PDB structure with 1.67 Å resolution), was selected (Table S3). To choose the best targets, the Uniport server (Table S4) and the Protparam server (Table S5) examined some important properties of proteins.

Subcellular locations and positions of mutagenesis were received from the Uniport server. All three targets have many mutations that affect each other’s pathways. Furthermore, the cellular locations of the Bax, Bcl2, and p53 are primarily located in the mitochondria, cytoplasm, cytosol, and nucleus. Moreover, ProtParam investigated various physical and chemical parameters [59]. The isoelectric point (pI) for p53 was more than 7; thus, p53 computed alkaline characteristics, while Bax and Bcl2 isoelectric were less than 7 which computed acidic characteristics. Additionally, negatively (ASP + GLU) and positively (ARG + LYS) charged residues were different in the three proteins. For p53, (ASP + GLU) was 43, and (ARG + LYS) was 45. Bcl2 had 22 (ASP + GLU) and 21 (ARG + LYS), while Bax had 23 (ASP + GLU) and 20 (ARG + LYS).

#### 3.1.2. Validation of Bax, Bcl2, and p53 Proteins

The 6EB6, 6GL8, and 6SL6 proteins were assessed by PROCHECK, Verify3D, and Ramachandran plots. ERRAT evaluates protein models determined by X-ray crystallography. The amount of ERRAT depends on the nonbonded atomic interactions in the protein structures [60]. The ERRAT scores of 6EB6, 6GL8, and 6SL6 were calculated as 99.4152%, 100%, and 88.1443%, respectively. In all three models, more than 94% of the amino acid residues had a 3D–1D score ≥ 0.2, indicated by Verify3D (Table S6). Moreover, the ProSA-web was valued at −6.76, −7.99, and −6.18 for 6EB6, 6GL8, and 6SL6, respectively. A Ramachandran plot obtains insight into the structure of proteins by the investigation of torsional angles [61]. In all three Ramachandran plots, no amino acid residues were recognized to be in the disallowed and generosity-allowed regions. These scores express accepted PDB structures.

#### 3.1.3. ADME Prediction

Lipinski rules and 24 ADMET properties were evaluated. Cur has a molecular weight of 368.38 g/mol and a log P value of 3.03, and contains two hydrogen bond donor atoms and six hydrogen bond acceptor atoms; therefore, Cur was accepted based on Lipinski’s rules (Table S7). In Table S8, physicochemical features such as (log P, log S, log D) were investigated. Cur’s log P (lipophilicity descriptor) is more than 3, demonstrating poor aqueous solubility and high membrane permeability. The quantity of log S is between −4 and −6, which is considered moderately soluble. It is closely related to Cur’s log P and log D, which rarely produce poor aqueous solubility and high membrane permeability. Human intestinal and epithelial colorectal adenocarcinoma absorption cells are in the correct range.

A significant attribute of the intestinal epithelium is the transporting back (efflux) of some substances that enter the cytoplasm of mucosal cells to the intestinal lumen by P-glycoprotein (P-gp), as a plasma membrane drug efflux transporter [62]. Cur is not removed from cells after internalization since it is not a P-gp substrate. The P-gp is involved in mediating multidrug resistance via drug efflux. P-gp identifies and transports a wide range of drugs. Therefore, the generation of potent P-gp inhibitors is important in preventing drug resistance development in cancer chemotherapy [63]. Table S8 indicated that Cur is a noninhibitor drug.

The extent of plasma protein binding (PPB) is vital for predicting exposure in pharmacokinetic modeling [64]. Plasma protein binding and drugs have hydrophobic and electrostatic interactions such as van der Waals and hydrogen bonds. These reversible interactions influence the drug’s volume of distribution, clearance, elimination, and pharmacological effect [65]. Regarding the mentioned physicochemical features of Cur and the percentage of PPB, approximately 87%, Cur has a high affinity for protein binding. Moreover, blood plasma consists of 92% water; therefore, Cur is hydrolyzed in aqueous plasma. Consequently, it possesses a low therapeutic index with high degradation.

According to the data, in the human plasma, the concentration of Cur after high oral administration (10 to 12 g per day) is in the nanomolar range [66]. Moreover, Cur can pass through the blood–brain barrier (BBB). However, several clinical trials about Cur’s potential in crossing the BBB failed due to low brain bioavailability [67]. Human volume distribution (VD) is essential for identifying human dose [68]. The low volume distribution showed that Cur was confined to blood and bound to plasma protein. Drug–drug interactions mediated by cytochrome P450 (CYP) inhibition cause drug adverse reactions. CYPs are membrane-bound enzymes mostly located in the mitochondria of hepatocytes, the smooth endoplasmic reticulum, and the intestines. Among the 57 CYP isoforms, 5 CYPs (CYPs 3A4, 2D6, 2C19, 2C9, and 1A2) metabolize approximately 80% of clinical drugs. The metabolism part indicated that Cur is a noninhibitor and nonsubstrate of CYP1A2, CYP2D6, CYP2C19, and CYP3A4; however, it is a CYP2D6 substrate and can inhibit its function [69,70,71]. Notably, Cur–Cur interactions caused by CYP2D6 inhibition remain long after passing through the liver and even long after drug discontinuation.

Cur revealed a rapid systemic clearance rate and a low half-life. Most of the Cur (60–70%) undergoes fast clearance and is excreted in the feces [72]. Cur blocks human Ether-à-go-go-Related Gene (hERG) potassium channel to prevent different types of cardiovascular toxicity [73]. However, human hepatotoxicity is one of the drawbacks of the Cur drug.

#### 3.1.4. Molecular Docking

**Figure 1:**
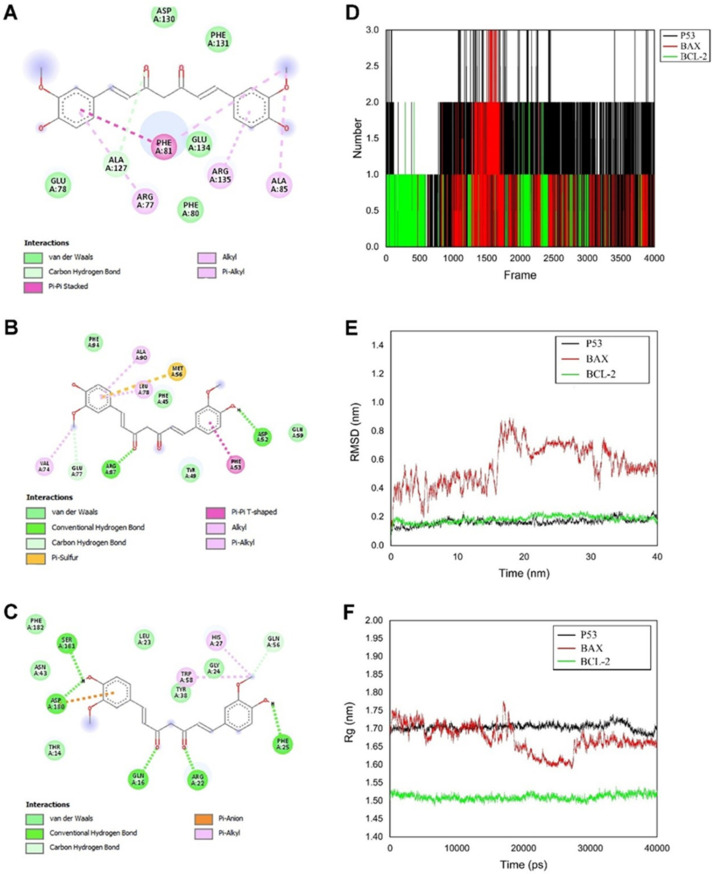
Interaction of ligands and target receptors. (**A**) Curcumin-Bax, (**B**) Curcumin-Bcl2, (**C**) Curcumin-p53; (**D**) Numbers of intermolecular hydrogen bonds in curcumin complex with p53 (black), Bax (red), and Bcl2 (green); (**E**) RMSD plot of curcumin complex with p53 (black), Bax (red), and Bcl2 (green); (**F**) The radius of gyration for p53 (black), Bax (red), and Bcl2 (green) in the presence of curcumin.

As a substantiate protocol, proteins were chosen according to binding affinity <−5 and RMSD < 2. Cur generated nine conformers from molecular docking, and only one of the conformers for all three targets has an ideal binding affinity and RMSD. Molecular-docking studies of Bax, Bcl2, and p53 with Cur are shown in (Table S9). Furthermore, Figure 1A–C manifested the amino acid residues which participated in interactions between Cur and macromolecules.

#### 3.1.5. Hydrogen Bonds

One of the significant analyses showing the stability of the ligand–protein complex is the analysis of the number of hydrogen bonds. For this purpose, the number of hydrogen bonds formed between Cur and the modeled proteins (p53, Bax, and Bcl2) with the help of VMD (v1.9.3) was calculated [74]. The number of hydrogen bonds between Cur and proteins concerning frames is illustrated in (Figure 1D). The Cur formed 32, 17, and 23 unique hydrogen bonds with p53, Bax, and Bcl2, respectively. As can be seen, p53 and Bax formed a maximum of three bonds with Cur, while Bcl2 formed a maximum of two bonds. Additionally, it is noticeable that p53 and Cur formed two hydrogen bonds most of the time, but Bax formed one hydrogen bond. These results confirm our docking study. It can be concluded that the Cur complex with p53 is more stable than the Bax and Bcl2 complex. Additionally, the residues which engage in hydrogen with Cur are reported in Table S10. For further study, we also analyzed the donor–acceptor distance, in which we found that Cur can form hydrogen bonds with approximately 0.35 nm distance (0.39 ± 0.28 and 0.38 ± 0.23 nm) with p53. At the same time, this value was 0.57 ± 0.26 nm and 0.68 ± 0.18 nm for Bax and Bcl2, respectively. We can conclude that the bond in p53 is more favorable than in the Bax and Bcl2 one.

#### 3.1.6. Root Mean Square Deviation of Atomic Positions (RMSD)

RMSD is a way to predict conformational change and evaluate the stability of a protein. The RMSD plot of p53, Bax, and Bcl2 proteins with Cur is illustrated in Figure 1E. There are no significant fluctuations in the RMSD of the p53 and Bcl2 complex; although, in the first time of the simulation trial, these proteins experience the initial kinetic shock [75]. Our findings suggest that p53 and Bcl2 have stable complexes. The RMSD curve for Bax shows significant change, which means the Cur effects the protein shape.

#### 3.1.7. Radius of Gyration

The compactness of a protein throughout simulation can be analyzed using the radius of gyration (Rg) property. A stable Rg shows a stably folded protein, and a change in Rg means an unfolding in the protein [76]. Figure 1F depicts the Rg of modeled proteins (p53, Bax, and Bcl2) in the presence of Cur. The value of Rg for our proteins was approximately 1.52 nm for Bcl2, 1.65 nm for Bax, and 1.70 nm for p53. As seen in Figure 1F, the radius of gyration of p53 and Bcl2 in the complex with Cur had no fluctuation, and this shows that these proteins were stably folded, i.e., could stably fold to each other. The Bax first had an Rg of 1.70 nm value, but in nearly 20–30 ns, the value of Rg dropped and then increased to its first value and was stable up to the end of the simulation. This change is likely due to the dynamic nature of this protein. Despite all the advantages of Cur as a drug candidate against cancer, the intrinsic Cur characteristics include low bioavailability, poor water solubility (which leads to poor absorption), degradation and high metabolic rate, chemical instability, and short half-life, which are substantial obstacles to its use for cancer treatment. Novel nanodrug delivery approaches have emerged and hold great potential to improve the therapeutic ability of curcumin.

### 3.2. Fabrication and Characterization

#### 3.2.1. Particle Size

**Figure 2:**
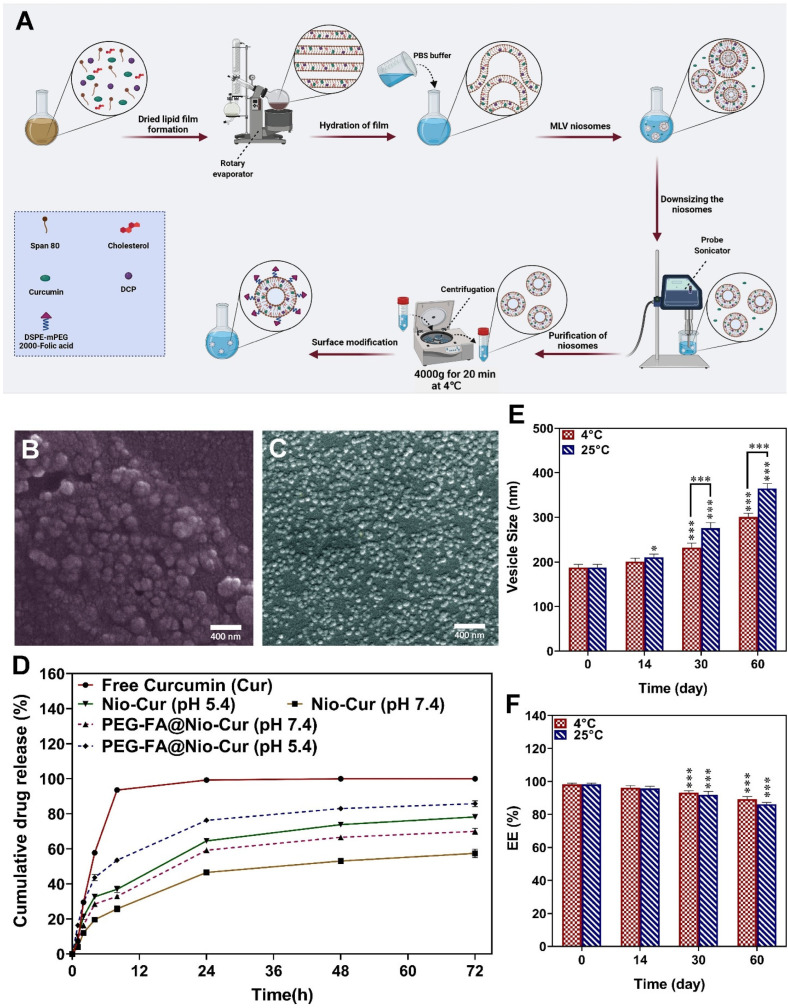
(**A**) The preparation of functionalized niosomes by the thin-layer hydration method. MLV: multilamellar vesicles; SUV: small unilamellar vesicles; (**B**) SEM image of the prepared optimized Nio-Cur3; (**C**) SEM image of the prepared optimized PEG-FA@Nio-Cur3; (**D**) In vitro release of Cur from Nio-Cur3 and PEG-FA@Nio-Cur3 at pH 7.4 and pH 5.4 in 37 °C; (**E**) Size stability evaluation of PEG-FA@Nio-Cur3 and (**F**) EE (%) stability evaluation of PEG-FA@Nio-Cur3 after two months of storage at 4 ± 2°C and 25 ± 2 °C; Data are represented as mean ± SD and *n* = 3; *p* < 0.001 ***, *p* < 0.05 *.

Nio-Cur was prepared using the thin-film hydration method (Figure 2A). The average size of the niosomal formulations is shown in Table S11. The results indicate that the amount of the surfactant HLB and the drug: lipid molar ratio affected the size of vesicles. The observed effects for span 20 (HLB 8.6), span 60 (HLB 4.7), and span 80 (HLB 4.3) with a similar drug: lipid molar ratio (10) reveal that an increase in the HLB of the surfactant resulted in a relevant increase in the size of the vesicles because the largest vesicles were composed of span 20 (333.20 ± 21.07) while the smallest vesicles were prepared from span 80 (218.30 ± 12.41). The findings obtained for span 20 (HLB 8.6), span 60 (HLB 4.7), and span 80 (HLB 4.3) with a similar drug: lipid molar ratio 20 show that niosomal formulations with a lipid: drug ratio of 10 had smaller structures compared to similar formulations containing a lipid: drug ratio of 20. Vesicles prepared from span 80 with a drug: lipid molar ratio of 10 were smaller than samples composed of span 80 with a drug: lipid molar ratio of 20, 218.30 ± 12.41, and 221.50 ± 13.31, respectively. Among the coated and uncoated Nio-Cur 3 formulations, PEG-FA@Nio-Cur 3 had the lowest average particle size (187.13 ± 7.55 nm). The vesicle size distribution of formulas formed from different surfactants was not narrower than vesicles coated with PEG-FA since the PDI (polydispersity index) values were more significant (0.25 ± 0.009–0.39 ± 0.016) than the PDI value of PEG-FA-Nio-Cur 3 (0.160 ± 0.033), as presented in Table S11.

#### 3.2.2. Entrapment Efficiency

The amount of lipid: drug molar ratio and type of surfactant used in the formulation preparation affected the encapsulation efficiency or EE% of the niosome. The EE% values achieved for span 20, span 60, and span 80 with a similar lipid: drug ratio of 10 followed the trend span 20 (97.8778 ± 0.2962) > span 60 (96.7111 ± 0.4194) > span 80 (92.7111 ± 0.3469) (Table S11). The EE% of vesicles formed from span 20, 60, and 80 with a lipid: drug ratio of 20 was 95.7667 ± 1.3017, 94.9944 ± 0.0096, and 91.8222 ± 0.0962, respectively. PEG-FA@Nio-Cur 3 had the largest EE% compared to the other niosome formulations. The EE% of PEG-FA@Nio-Cur 3 and Nio-Cur 3 was 98.2517 ± 0.7851 and 96.7111 ± 0.4194, respectively. Coating the niosome with PEG affected the EE% of formulations.

#### 3.2.3. Size Distribution, Zeta Potential, and Morphology of the Nanoformulations

The structure and surface morphology of Nio-Cur3 and PEG-FA@Nio-Cur3 were analyzed using SEM. Field emission–SEM images of the optimized formulations for PEG-FA@Nio-Cur3 and Nio-Cur3 are shown in Figure 2B,C. The vesicles made of PEG-FA@Nio-Cur3 had a more regular globular morphology and smooth surface than Nio-Cur3 alone. Moreover, the vesicle size of PEG-FA@Nio-Cur3 (50 nm) was smaller than that of the Nio-Cur3 vesicle (60–70 nm). According to the SEM imaging results, the optimum formulation of PEG-FA@Nio-Cur3 shows a more spherical shape, homogeneous throughout the samples, than Nio-Cur3. In the DLS (dynamic light scattering) method, the size of the nanoparticles was analyzed based on hydrated vesicles. In contrast, the size of nanoparticles in the SEM method was studied in dried forms (measuring the exact diameter of each particle, expressed in terms of the Z-average). According to the previous reports, the dry vesicles in the dried dispersion were smaller than the hydrated vesicles in the diluted dispersion, i.e., the dried diameters were smaller than the hydrodynamic diameters.

TEM was employed to evaluate the morphology of optimum niosomes. Figure S3A,B illustrate the internal structure of Nio-Cur and PEG-FA@Nio-Cur, respectively. The size of Nio-Cur (Figure S3A) and PEG-FA@Nio-Cur (Figure S3B) is approximately 60–70 nm and 40–50 nm, respectively. The image shows the two-layered and spherical shape of optimum niosomes. Furthermore, the rigid boundaries of the niosomes’ structure can be seen.

The zeta potential of Nio, Nio-Cur, and PEF-FA@Nio-Cur in the optimum formulation was obtained as −28.7 mV, −22.6 mV, and −8.1 mV, respectively (Figure S1).

#### 3.2.4. Drug Release and Modeling Kinetic

Figure 2D shows the studies of Cur release from the prepared formulations (Nio-Cur3 and PEG-FA@Nio-Cur3). The release from niosomes was measured for 72 h in PBS-SDS medium under physiological (~7.4) and pathological cancerous (~5.4) pH conditions. As seen, Cur showed an initial burst release period for 8 h; additionally, the release rates of Cur from Nio-Cur 3 (optimum formulation of curcumin-loaded niosome) and PEG-FA@Nio-Cur 3 in physiological pH (pH ~7.4) were significantly lower than that of uncoated and coated niosomes in pathological cancerous pH (pH ~5.4). These results show that the drug release was pH-dependent. After the first phase of fast release, which is related to the drug’s diffusion from the outer layers of the niosomes, all formulations showed a phase of slower release up to stabilization at 72 h. As shown in Figure 2D, the Cur release rate of Nio-Cur was less than that of free Curcumin. The release data indicate that Nio-Cur3 had a lower release rate than PEG-FA@Nio-Cur3 at pH 7.4 and 5.4. In the Nio-Cur 3 formulation, at pH = 7.4, the drug release in 72 h was about 58% and at pH = 5.4 was approximately 79%. In the FA@Nio-Cur3 formulation, at pH = 7.4, the drug release of Cur in 72 h was about 70% and at pH = 5.4, approximately 86%. However, at a lower pH, a higher increase was found in the release showing that PEG-FA@Nio-Cur3 may be more suitable for intracellular drug delivery and drug delivery in cancerous tissues with mildly acidic conditions in comparison with Nio-Cur 3. Moreover, the Cur release was temperature-dependent. At 25 °C (Figure S3), in the Nio-Cur 3 formulation, at pH = 7.4, the drug release in 72 h was about 47% and at pH = 5.4 was approximately 62%. In the FA@Nio-Cur3 formulation, at pH = 7.4, the drug release of Cur in 72 h was about 55% and at pH = 5.4, approximately 73%.

Table S12 lists the result of various release kinetics models obtained for all samples. The model that has the highest value of R2 is suggested as the best model for the release mechanism of Cur. So, based on the highest R2 value for curve fitting, the best-fitting model for free Cur was the first-order model (the rate of release depends on drug concentration). However, the Higuchi model in both temperatures was the best model for optimized Nio-Cur 3 at pH = 7.4 and Nio-Cur 3 at pH = 5.4. Additionally, the best release kinetics model for PEG-FA@Nio-Cur 3 at pH = 7.4 was Higuchi (25 °C and 37 °C) and PEG-FA@Nio-Cur 3 at pH = 5.4 was Korsmeyer–Peppas (25 °C and 37 °C). Then, *n* values obtained (0.43 < *n* < 0.85) denote Fickian diffusion drug release [56].

#### 3.2.5. Stability

The physical stability of PEG-FA@Nio-Cur3 and Nio-Cur 3 formulations was evaluated by characterization of vesicle size, PDI, and EE% after storage at 4 °C and 25 °C for 60 days. As shown in Figure 2E,F, an increase in the size and PDI and a decrease in EE% were observed during storage time because the leakage of the drug occurred for the niosomal formulations. According to the data for particle size, PDI, and EE%, the PEG-FA@Nio-Cur3 formulation was more stable than Nio-Cur3. In the PEG-FA@Nio-Cur3 niosomes (Figure 2F,E and Figure S4D), EE%, PDI, and size changed from 98% to 87%, 0.17 to 0.27, and 197 nm to 297 nm at 4 °C after 60 days. However, in Nio-Cur3 (Figure S4A–C), EE%, PDI, and size changed from 98% to 96%, 0.2 to 0.37, and 210 nm to 397 nm at 4 °C after 60 days.

#### 3.2.6. Cell Proliferation

**Figure 3:**
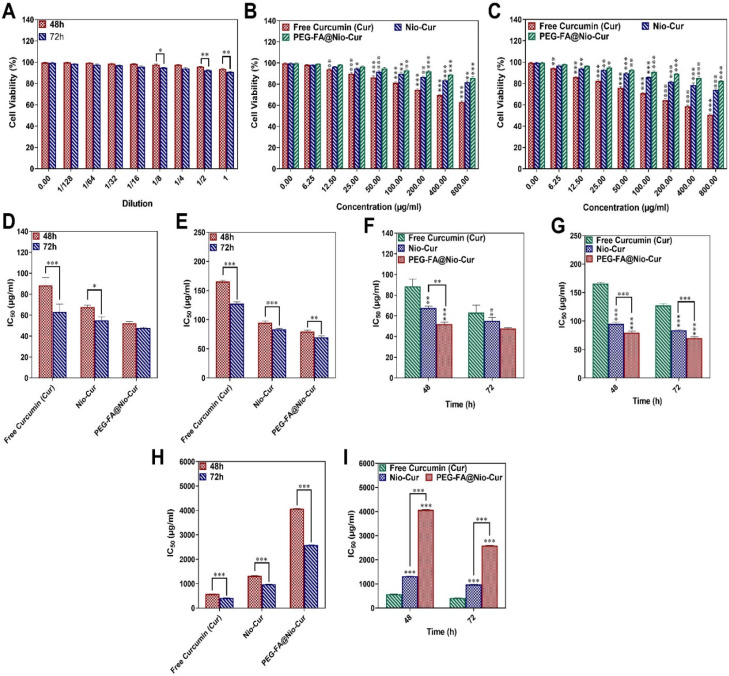
(**A**) Percentage cell viability of different dilutions of vehicle (Nio) on nonmalignant MCF10A cells; (**B**) The effects of Cur, Nio-Cur, and PEG-FA@Nio-Cur on the viability of MCF10A cells in 48 h; (**C**) The effects of Cur, Nio-Cur, and PEG-FA@Nio-Cur on the viability of MCF10A cells in 72 h; (**D**) Half-maximal inhibitory concentration (IC50) values after 48 h and 72 h exposure of MCF7 breast cancer cells to Cur, Nio-Cur, and PEG-FA@Nio-Cur; (**E**) Half-maximal inhibitory concentration (IC50) values after 48 h and 72 h exposure of 4T1 breast cancer cells to Cur, Nio-Cur, and PEG-FA@Nio-Cur; (**F**) Comparison of in vitro cytotoxic impacts of all samples in MCF7 breast cancer cells in 48 and 72 h; (**G**) Comparison of in vitro cytotoxic impacts of all samples in 4T1 breast cancer cells in 48 and 72 h. (**H**) Half-maximal inhibitory concentration (IC50) values after 48 h and 72 h exposure of MCF10A cells to Cur, Nio-Cur, and PEG-FA@Nio-Cur; (**I**) Comparison of in vitro cytotoxic impacts of all samples in MCF10A cells in 48 h and 72 h. Data represent means ± standard deviations (*n* = 3). For all charts, ***: *p* < 0.001; **: *p* < 0.01; *: *p* < 0.05.

It was found that serial dilutions of empty niosomes had no effect on the viability of MCF10A cells (nonmalignant breast epithelial cells) statistically (*p* < 0.05; Figure 3A), except at 1/8, 1/2, and 1/1 dilution. In examining the effects of different concentrations of free Cur, Nio-Cur, and PEG-FA@Nio-Cur on the nonmalignant MCF10A cell line after 48 h treatment, the cell viability of MCF10A cells significantly decreased (*p* < 0.05) in the concentrations of 25, 50, 100, 200, 400, and 800 μg/mL compared to the control group in three formulations (Figure 3B). In examining the effects of different concentrations of free Cur, Nio-Cur, and PEG-FA@Nio-Cur on the nonmalignant MCF10A cell line after 72 h treatment, the cell viability of MCF10A cells significantly decreased (*p* < 0.05) in the concentrations of 100, 200, 400, and 800 μg/mL compared to the control group in three formulations (Figure 3B).

MCF7 breast cancer cells were treated with different formulations, and their effects on the survival of MCF7 breast cancer cells after 48 and 72 h are summarized in Figure 3D. Respectively, the IC50 (half-maximal inhibitory concentration) values of all tested formulations after 48 and 72 h were: Cur (88.45 ± 7.31 and 63.12 ± 7.31 µg/mL), Nio-Cur (67.69 ± 1.80 and 55.17 ± 3.30 µg/mL), and PEG-FA@Nio-Cur (52.20 ± 1.71 and 47.75 ± 0.82 µg/mL). There were significant decreases in the IC50 values of Cur and Nio-Cur after 72 h compared with 48 h treatment (*p* < 0.001 and *p* < 0.05, respectively). The experimental results also showed that PEG-FA@Nio-Cur induced no significant decrease in the IC50 values after 72 h compared with 48 h. As shown in Figure 3F, the IC50 values of all formulations were compared each time separately (48 and 72 h). After 48 h, there were significant decreases in the IC50 values of Nio-Cur and PEG-FA@Nio-Cur compared to the control sample (free Cur; *p* < 0.01 and *p* < 0.001, respectively), and the IC50 values of PEG-FA@Nio-Cur significantly decreased compared to Nio-Cur (*p* < 0.01). After 72 h, there were significant decreases in the IC50 values of Nio-Cur compared to free Cur (*p* < 0.01), and a significant decrease was observed in the IC50 values of PEG-FA@Nio-Cur compared to Nio-Cur (*p* < 0.01); moreover, statistically significant changes were not observed between PEG-FA@Nio-Cur with Cur and PEG-FA@Nio-Cur with Nio-Cur.

4T1 breast cancer cells (derived from the mammary gland of a BALB/c mouse) were treated with different formulations, and their effects on the survival of 4T1 breast cancer cells after 48 and 72 h are summarized in Figure 3E. Respectively, the IC50 values of the different formulations after 48 and 72 h were: Cur (165.67 ± 2.02 and 127.55 ± 3.35 µg/mL), Nio-Cur (95.09 ± 2.75 and 83.72 ± 1.50 µg/mL), and PEG-FA@Nio-Cur (79.53 ± 2.71 and 69.88 ± 2.70 µg/mL). There were significant decreases in the IC50 values of Cur, Nio-Cur, and PEG-FA@Nio-Cur after 72 h compared with 48 h treatment (*p* < 0.001, *p* < 0.001, and *p* < 0.01, respectively). As shown in Figure 3G, the IC50 values of all formulations were compared each time, separately (48 and 72 h). After 48 h, there were significant decreases in the IC50 values of Nio-Cur and PEG-FA@Nio-Cur compared to free Cur (*p* < 0.001), and there were significant decreases in the IC50 values of PEG-FA@Nio-Cur compared to Nio-Cur (*p* < 0.001). After 72 h, there were significant decreases in the IC50 values of Nio-Cur and PEG-FA@Nio-Cur compared to free Cur (*p* < 0.001) and a significant decrease was observed in the IC50 values of PEG-FA@Nio-Cur compared to Nio-Cur (*p* < 0.001). The results of cell toxicity for each formulation at different times are reported in the Supplementary Materials (Figure S5).

#### 3.2.7. Apoptosis

**Figure 4:**
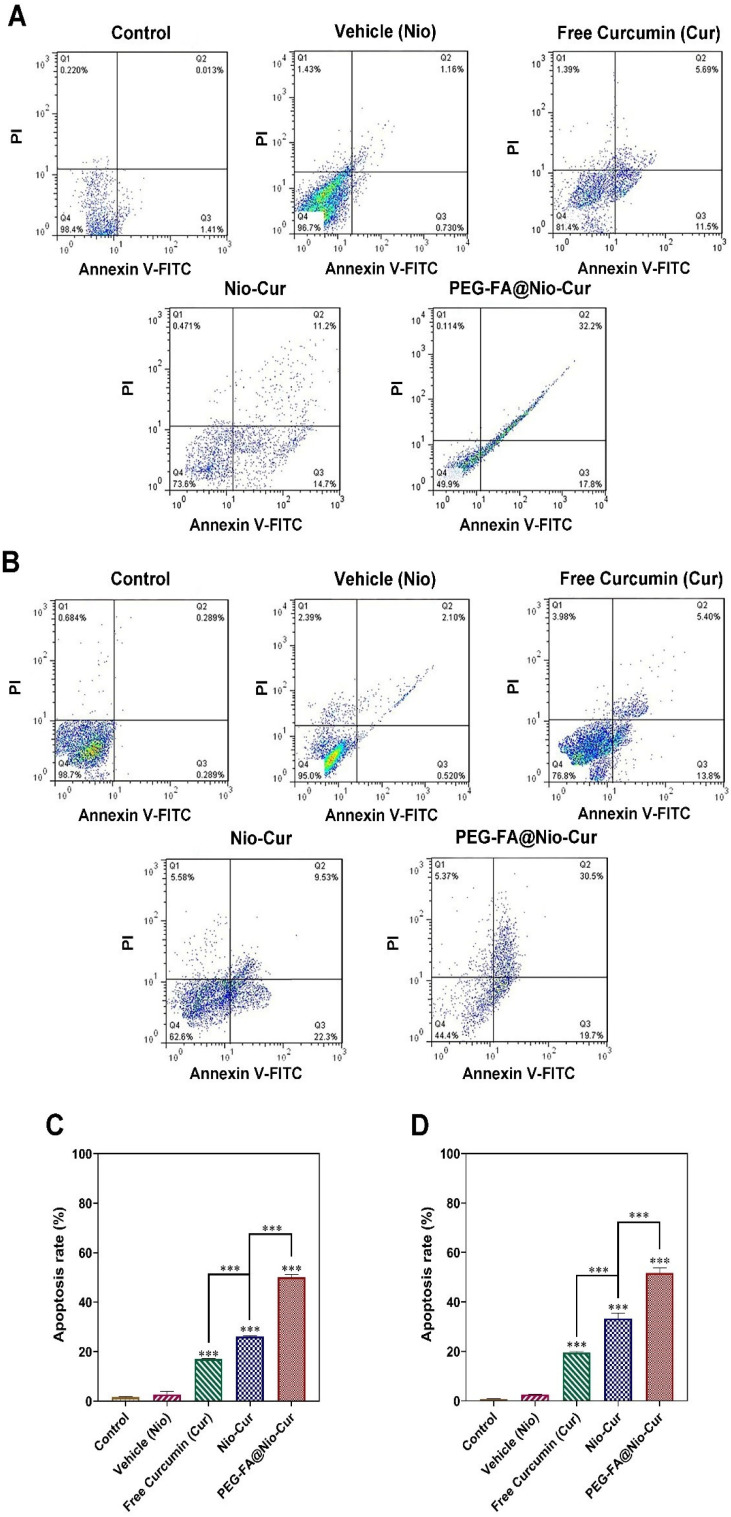
The effects of control, Cur, and different niosome formulations on vehicle (Nio), Nio-Cur and PEG-FA@Nio-Cur of MCF7 and 4T1 breast cancer cells that had undergone 48 h of treatment are shown in (**A**,**B**), respectively. Means and standard deviations are represented by the data (*n* = 3). For all charts, ***: *p* < 0.001; **: *p* < 0.01; *: *p* < 0.05. Flow cytometric analysis of (**C**) MCF7 and (**D**) 4T1 cells after treatment with IC50 concentration of vehicle (Nio), Cur, Nio-Cur, and PEG-FA@Nio-Cur formulations. Nonmalignant breast epithelial cell line MCF10A did not show any statistically significant alterations in survival after being exposed to serially diluted empty niosomes at various concentration levels.

After double staining of the cells with annexin V-FITC (fluorescein thiocyanate-labeled annexin V) and PI (propidium iodide), apoptosis of breast cancer cells (MCF7 and 4T1 cells) was measured quantitatively with flow cytometry. The administration of Nio, Free Cur, Nio-Cur, and PEG-FA@Nio-Cur to the MCF7 cells (Figure 4A) and the 4T1 cells (Figure 4B) induced apoptosis of both types of breast cancer cells. The percentages of apoptotic cells in both cell lines (Figure 4C,D) that were treated with Nio were not significantly different from the control cells that were not exposed to any of the niosomal formulations (*p* > 0.05). The percentage of apoptotic MCF7 breast cancer cells was significantly increased over the control after the cells were exposed to Free Cur, Nio-Cur, and PEG-FA@Nio-Cur (*p* < 0.001). The percentages of apoptotic MCF7 cells after treatment with PEG-FA@Nio-Cur significantly increased compared to those treated with Nio-Cur (*p* < 0.001; Figure 4C). On the contrary, the percentage of apoptotic cells decreased when Free Cur was used instead of the Nio-Cur or PEG-FA@Nio-Cur (*p* < 0.001). Similar trends were identified for the 4T1 murine breast cancer cells (Figure 4D). In total, comparisons indicated that the anticancer effect of PEG-FA@Nio-Cur on the two cell lines was higher than in the other groups and reached its highest value compared to other groups.

#### 3.2.8. Cell Cycle

**Figure 5:**
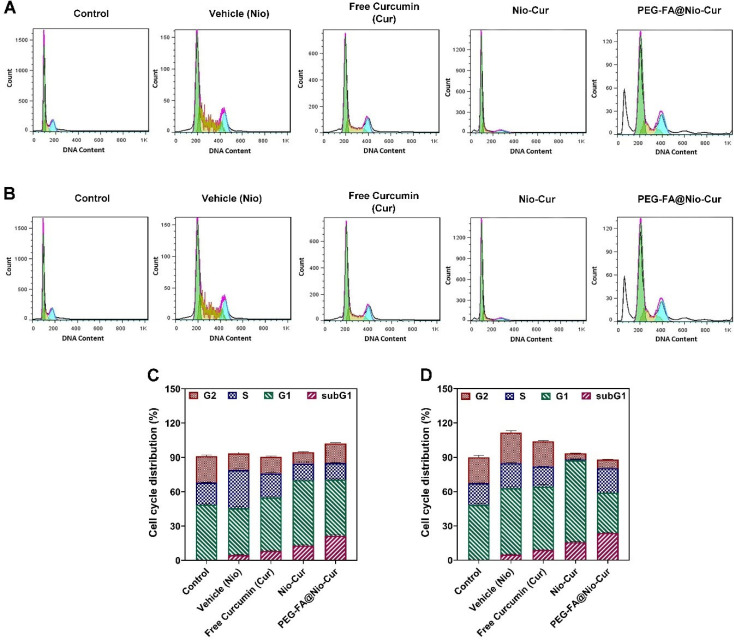
The effects of control, Cur, and different niosome formulations vehicle (Nio), Nio-Cur and PEG-FA@Nio-Cur of (**A**) MCF7 and (**B**) 4T1 breast cancer cells on cell cycle distribution after 48 h of treatment. Data represent means ± standard deviations (*n* = 3). (**C**,**D**) Cell cycle distribution for (**C**) MCF7 and (**D**) 4T1 cells after treatment with IC50 concentration of vehicle (Nio), Cur, Nio-Cur, and PEG-FA@Nio-Cur formulations.

All cells enter the following stages during their cycle: G1, S, G2, and M. The cells affected by antimitosis compounds do not leave the gap 1 (G1) phase. The effects of niosomes, free Curcumin, Nio-Cur, and the PEG-FA@Nio-Cur formulations on the MCF7 and 4T1 cells during their cycle were examined by flow cytometry. As Figure 5C cell cycle-MCF7 and Figure 5D cell cycle-4T1 demonstrate, the sustained effect of PEG-FA@Nio-Cur was manifested by cells turning to a sub-G1 phase for each tested cell line (MCF7 cells: 12.79 ± 0.66% for Nio-Cur and 21.31 ± 1.13% for PEG-FA@Nio-Cur; 4T1 cells: 15.67 ± 0.65 for a Nio-Cur and 23.64 ± 0.76 for PEG-FA@Nio-Cur). Moreover, the proportion of the cells in the different cell cycle phases is listed in Figure 5A,B.

#### 3.2.9. Quantitative Real-Time Polymerase Chain Reaction (qRT-PCR)

**Figure 6:**
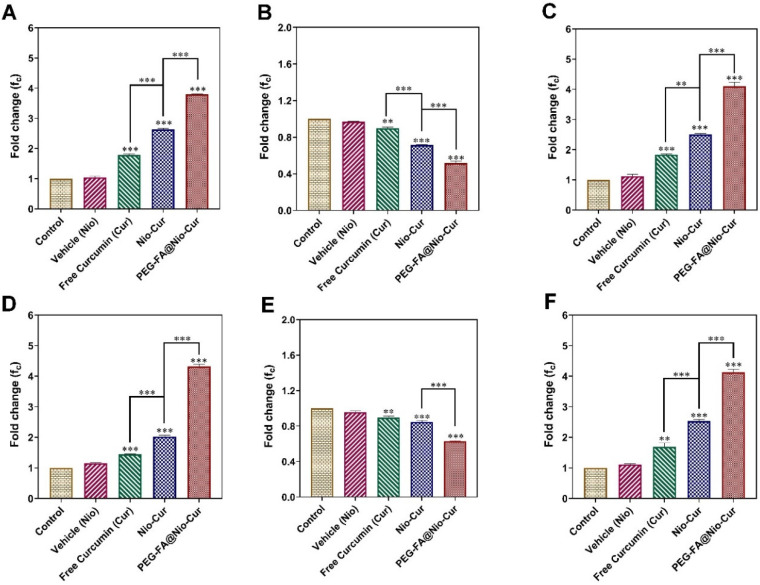
(**A**) Expression levels of Bax, (**B**) Bcl2, and (**C**) p53 genes in MCF7 cells and (**D**) Expression levels of Bax, (**E**) Bcl2, and (**F**) p53 genes in 4T1 cells after those breast cancer cells were exposed to Vehicle (Nio), Free Curcumin, Nio-Cur, and PEG-FA@Nio-Cur (β-actin as housekeeping gene). Data represent means ± standard deviations (*n* = 3). For all charts, ***: *p* < 0.001; **: *p* < 0.01; *: *p* < 0.05.

Due to the key role of Bcl2 and Bax proteins in apoptosis, the expression patterns were investigated in the treated cancerous cells. Figure 6A to F register the RT-PCR results for expressing Bcl2, Bax, and p53 genes at transcriptional levels (forward and reverse primer sequences for the aforementioned genes are listed in Table S2). In the MCF7 cell line, after 48 h of exposure to the Cur, Nio-Cur, and PEG-FA@Nio-Cur, a significant increase in the expression level of the proapoptotic Bax gene was observed (Figure 6A). Meanwhile, as shown in Figure 6B, the expression level of the antiapoptotic Bcl2 gene significantly decreased. Moreover, the expression level of p53 increased significantly (Figure 6C). In the 4T1 cell line, after 48 h exposure to the drug-loaded niosomes, a significant increase in the expression level of the proapoptotic Bax gene was observed (Figure 6D). Meanwhile, as shown in Figure 6E, the expression level of the antiapoptotic Bcl2 gene significantly decreased. Moreover, the expression level of p53 increased significantly (Figure 6F). Exposing tumor cells to Cur, Nio-Cur, and PEG-FA@Nio-Cur induced apoptosis via enhancing the Bax: Bcl2 ratio. In both cell lines, a significant increase in the expression of these proapoptotic genes and a significant decrease in the expression of antiapoptotic genes (p53) were observed after exposure to PEG-FA@Nio-Cur, compared to Nio-Cur formulation (*p* < 0.001). Figure S6A–F register RT-PCR results for the expression of Bcl2, Bax, and p53 genes with PBGD as a housekeeping gene. In both cell lines, after 48 h exposure to the Cur, Nio-Cur, and PEG-FA@Nio-Cur, a significant increase (*p* < 0.001) in the expression level of the proapoptotic Bax and p53 gene were observed (Figure S6A,C,D,F). Mean-while, as shown in Figure S6B,E, the expression level of the antiapoptotic Bcl2 gene significantly decreased (*p* < 0.001).

#### 3.2.10. Reactive Oxygen Species

**Figure 7:**
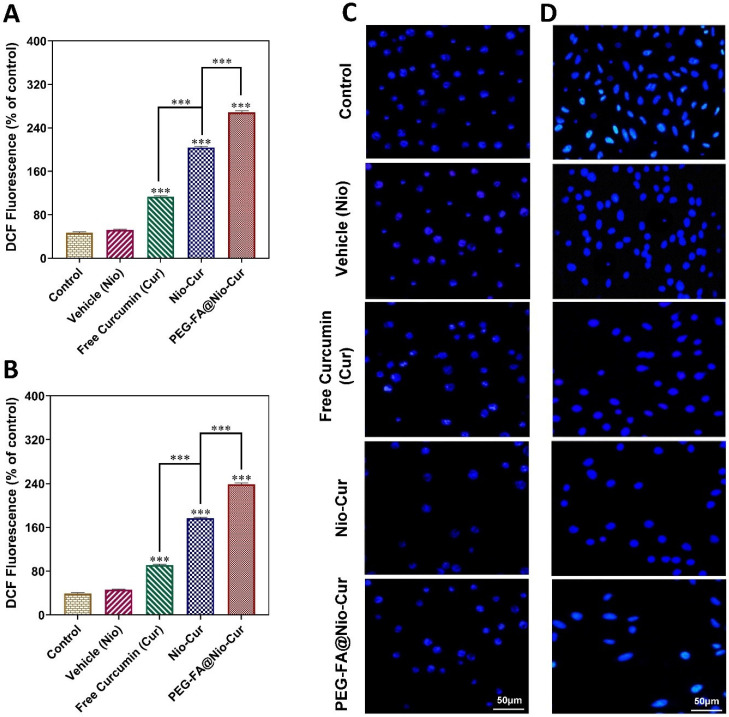
(**A**,**B**) Changes in intracellular ROS content, indicated by the fluorescence of 2′,7′-dichlorodihydrofluorescein (DCF), are summarized in (**A**) for MCF7 cells and (**B**) for 4T1 cells; (**C**,**D**) DAPI staining of vehicle (Nio), Cur, Nio-Cur, and PEG-FA@Nio-Cur on (**C**) MCF7 and (**D**) Fluorescence in 4T1 is brighter than in normal nuclei because of the apoptotic condition of compacted chromatin (control). Data represent means ± standard deviations (*n* = 3). For all charts, ***: *p* < 0.001; **: *p* < 0.01; *: *p* < 0.05.

The basic levels of reactive oxygen species (ROS) appear vital for cancer cell growth. However, ROS overgeneration can stimulate cell death [77]. Compared to the control group, MCF7 cells that were exposed to Free Cur, Nio-Cur, and PEG-FA@Nio-Cur showed significant increases in the fluorescence of 2′,7′-dichlorodihydrofluorescein (DCF), a fluorescent probe for measuring ROS (*p* < 0.001; Figure 7A). The DCF fluorescence of MCF7 human breast cancer cells treated with PEG-FA@Nio-Cur was significantly augmented compared with cells exposed to Nio-Cur (*p* < 0.001). Additionally, compared with the Free Cur, the Nio-Cur group enhanced the DCF fluorescence (*p* < 0.001). These results were similar to the results of 4T1 murine breast cancer cells (Figure 7B).

#### 3.2.11. Cellular Uptake and DAPI Staining

**Figure 8:**
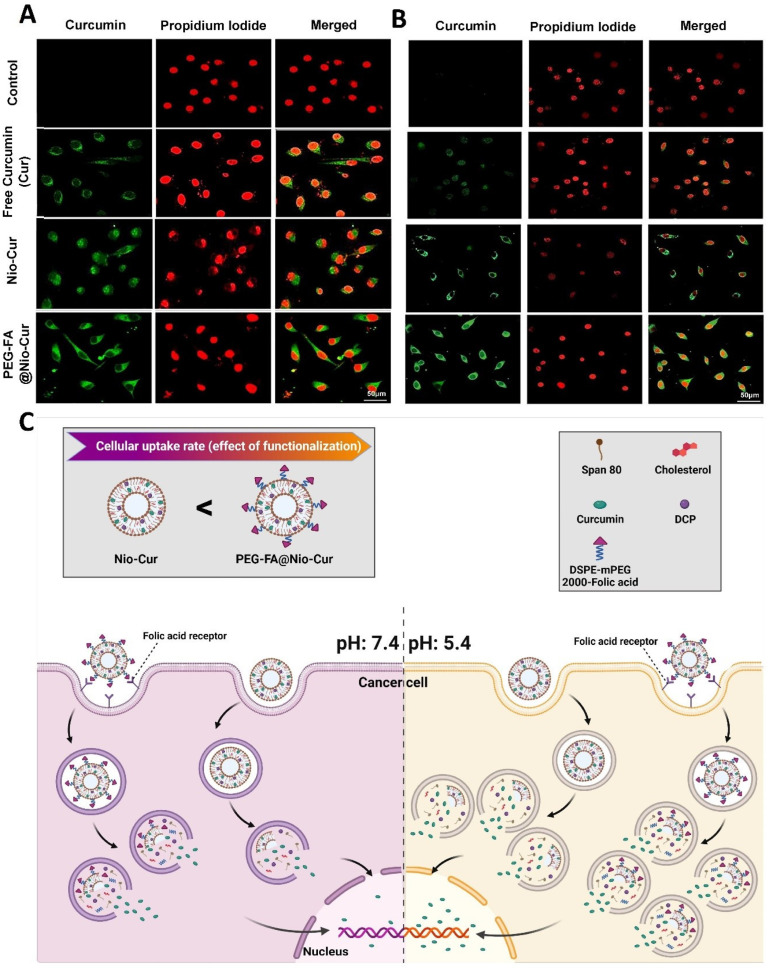
(**A**,**B**) Uptake of niosomes into MCF7 (**A**) and 4T1(**B**) cells was investigated with confocal laser scanning microscopy. The niosomes investigated were Cur, Nio-Cur, and PEG-FA@Nio-Cur; the nucleus was stained with propidium iodide (red). Curcumin shows green fluorescence; (**C**) schematic depicting the effect of pH on the release of contents from a niosome.

DAPI staining was performed in MCF7 and 4T1 cells treated with various formulations and compared with untreated controls. Fragmented or condensed chromatin are cells in the apoptotic phase features, which make nuclei show brighter fluorescence than normal nuclei. As shown in Figure 7C, folic acid conjugation improved the efficacy of PEG-FA@Nio-Cur in inducing nuclear condensation in MCF7 cells and enhanced the ability of curcumin to induce apoptosis. Additionally, in 4T1 cells, FA conjugation improved the efficacy of PEG-FA@Nio-Cur in causing nuclear condensation and enhanced the ability of Cur to induce apoptosis (Figure 7D). The folate receptor is a recognized biomarker for tumor cells due to its overexpression on most malignant cells [78]. By confocal microscopy, we analyzed the effects of FA on the cell adhesion of PEG-FA@Nio-Cur particles which will result in better drug delivery. The experiment shows that FA conjugation to PEG@Nio-Cur improved the internalization of PEG-FA@Nio-Cur compared to free Cur and Nio-Cur in MCF7 cells (Figure 8A). Additionally, the same effect was observed in treated 4T1 cells (Figure 8B). In addition, endocytosis was the most prominent in the cells exposed to PEG-FA@Nio-Cur, compared with cells exposed to Nio-Cur (Figure 8C).

## 4. Discussion

According to Table S1, the HLB of the surfactant and the drug: lipid molar ratio can affect the average size, PDI, and EE% of niosomes. This trend results from surface-free energy, decreasing with increasing hydrophobicity [79]. It has been reported that span 80-based niosomes were smaller than niosomes composed of sorbitan monoesters (span 20, 40, and 60) [80]. It was shown that the vesicle size of formulations increased with raising the amount of lipid (cholesterol), manifesting the properties of the physical stability of the vesicle and the rigidity of its membrane [53]. Another reason could be that cholesterol may be absorbed in an extended number of bilayers. It has also a limited impact on bilayer surfaces and separated interlayers [81,82,83]. PEG-FA@Nio-Cur had the smallest size of formulations. The EE% of the niosomes is also affected by the HLB value of surfactant [84]. As the HLB value of surfactant decreases from 8.6 to 1.7, EE% shows a decrease [85]. An increase in the lipid: drug ratio value results in decreasing EE%. The results also showed that increasing lipid concentration led to a significant decrease in EE%. This might be due to increased cholesterol in certain circumstances that disrupt the regular linear structure of the vesicle membrane, decreasing EE% [86,87].

The PEG-FA@Nio-Cur3 formulation had higher EE% than Nio-Cur. This result shows that the PEGylating of niosomal formulations is important in minimizing problems related to niosomal instability, such as drug leakage [88]. FESEM images of the optimized formulations showed the size of PEG-FA@Nio-Cur3 was smaller than Nio-Cur3. A decrease in particle size of the Nio-Cur3 formulations after using PEG modification can be attributed to providing the aqueous layer on the surfaces of niosomes through adding PEG. As a result, the steric hindrance prevents the aggregation of PEG-Nio formulation [89]. This reduction in aggregation would have resulted in the formation of niosomal formulations, which have a smaller size than naked ones. The drug release is because of the typical behavior of the niosomes in acidic conditions, which result in swelling and breaking the niosome.

Moreover, this can be attributed to the electrostatic interaction between the drug and the surfactant and an ionization state at physiological pH [51,56]. The higher drug release of PEG-FA@Nio-Cur3 compared to Nio-Cur3 is due to the absorption of Cur on the surface of the PEG layer and subsequent rapid drug release. Compounds simply entrapped within the aqueous part of a vesicular carrier show such conditions [90]. Moreover, since such compounds can interact with bilayer structures, drug permeation through bilayers and the retention phenomenon will happen because of the drug–bilayer compound interaction, increasing the carrier-loading capacity and drug release [91].

The stability result shows that the modification of niosomal formulations with PEG-FA plays an important role in minimizing problems related to niosomal instability, such as aggregation, fusion, and drug leakage [88]. In addition, the stability of samples stored at 4 °C is higher than samples stored at 25 °C in general. The reason is the higher stability of the hydrophobic noisome at low temperatures after storage at high temperatures, which leads to heterogeneous particle sizes and wide size distribution [92]. Another reason is that niosomal vesicles have lower mobility and permeability at 4 °C, although the niosome stored at 25 °C consists of larger vesicles.

This study investigated the cellular and molecular effects of curcumin loaded into PEG-FA-decorated niosomes against 4T1 and MCF7 cell lines. The treatment of MCF10A cells with free Cur, PEG-FA@Nio-Cur, and drug-free niosomes shows that the encapsulation of Cur by decorated niosomes leads to a remarkable improvement in the biocompatibility of the drugs, since these formulations are highly biocompatible with nonmalignant breast epithelial cells even at high concentrations. This biocompatibility seems to result from the low release rate of Cur from niosomes at the physiological pH (i.e., pH = 7.4); release data at this pH support the claim. As expected, the drug-free niosomes did not show any toxic effect on healthy cells, indicating they have enough biocompatibility to be used as a drug delivery system. Moreover, the treatment of breast cancer cell lines (4T1 and MCF7) with free Cur, Nio-Cur, and PEG-FA@Nio-Cur showed that the decoration of niosomes with FA resulted in higher cytotoxicity than other groups in the cancer cell lines. The DAPI staining assured the cytotoxicity mechanism as apoptosis. Interaction of the FA on the PEG-FA@Nio-Cur formulation with folate receptors on the surface of breast cancer cells can ease the internalization of the FA-decorated niosomes by receptor-mediated endocytosis and results in a higher uptake of PEG-FA@Nio-Cur compared to the other ones.

Excessively high amounts of ROS have been shown to cause cell death. ROS can constantly damage DNA, proteins, and lipids in biological systems when they are present. In addition to altering gene expression, redox receptor-binding proteins, redox enzyme-modifying enzymes, and protein turnover regulation, a high amount of ROS impairs the normal function of proteins [93,94]. Hypoxia, changes in nuclear and mitochondrial genes, activation of oncogenes, and the loss of tumor suppressor genes all cause cancer cells to create more ROS than normal cells. ROS are important for cancer cell proliferation, differentiation, and survival at low to moderate levels, but cause cell death at excessive levels. According to recent research, ROS may serve as a messenger in tumor cell invasion, angiogenesis, and metastasis [95,96]. In the present investigation, the amount of ROS in breast cancer cell lines treated with PEG-FA@Nio-Cur significantly increased compared to other groups. To evaluate whether the inhibition of breast cancer cells proliferation by free Cur, Nio-Cur, and PEG-FA@Nio-Cur was due to the apoptotic cell death, we determined the proportion of apoptotic cells after treatment of 4T1 and MCF7 cells with the aforementioned formulations. Tumor suppressor genes being lost, oncogenes being activated, and hypoxia all contributed to cancer cells producing more ROS than usual. Cancer cells require low to moderate ROS levels for growth, differentiation, and survival, but ROS cause cell apoptosis and death at excessive levels. According to recent studies, ROS may serve as a messenger in the invasion, angiogenesis, and metastasis of tumor cells. Flow cytometric analysis elucidated that the free Cur, Nio-Cur, and PEG-FA@Nio-Cur induce cytotoxicity by apoptosis stimulation in 4T1 and MCF7 cells. Moreover, treatment of cells with PEG-FA@Nio-Cur leads to a higher apoptosis rate than in other groups. Therefore, niosomal preparation did not affect the action mechanism of the drug. Duplication and division of eukaryotic cells occur in the cell cycle process. The probable DNA damage in each step of the cell cycle distinguishes by checkpoints and should be repaired before mitosis. Otherwise, cell apoptosis occurs [97,98].

The cell cycle study indicated that the loss of DNA content due to degradation in the presence of PEG-FA@Nio-Cur was higher; PEG-FA@Nio-Cur can place cancer cells in the subG1 phase (cell cycle figure). Therefore, the presence of PEG-FA@Nio-Cur in the vicinity of cancer cells can enhance the expression of Bax and p53 genes while declining Bcl2 expression. This change in gene expression pattern leads to DNA degeneration and apoptosis in cancer cells. The presence of PEG-FA@Nio-Cur in the cell enhances the p53 expression and this consequently leads to the increase in proapoptotic Bax gene expression and downregulation of antiapoptotic Bcl2 gene expression. The other groups had the same effect on cell apoptosis, but PEG-FA@Nio-Cur had the most severe effect among all groups. These results indicate that PEG-FA@Nio-Cur triggers mitochondrial-mediated apoptosis.

The progression of breast cancer cells relies on the balance between proapoptotic and antiapoptotic proteins (Bax to Bcl2 genes) [99,100]. Additionally, the qPCR study showed a considerable rise in the p53 protein’s expression level. By raising the expression of proapoptotic proteins (Bax and Bid) in the cell, p53 regulates the Bax: Bcl2 ratio. Moreover, the interaction between Bcl2 family proteins with p53 leads to activation of Bax and Bid and their translocation to the outer membrane of mitochondria. Moreover, to activate the mitochondrial pathway of apoptosis, p53 directly translocates to the mitochondria [101,102,103]. PEG-FA@Nio-Cur treatment of 4T1 and MCF7 breast cancer cells reduces antiapoptotic Bcl2 expression and increases the expression level of the Bax proapoptotic gene. Furthermore, Western blot analysis confirms these observations.

## 5. Conclusions

Our findings suggest that although Cur can be a good inhibitor for p53, Bax, and Bcl2, it can also cause a conformational change in Bax and has significant ADME drawbacks. The sizes of all the PEG-FA@Nio-Cur3 were <100 nm in diameter. Nio-Cur and PEG-FA@Nio-Cur3 were stored at a temperature of 4 and 25 °C, and the results demonstrated that PEG-FA@Nio-Cur3 formulation was more stable than Nio-Cur3. Moreover, the drug release in nanocarriers was sustained at pH = 7.4. However, the release was fast in acidic pH (~5.4). Since the PEG-FA@Nio-Cur3 formulation showed high performance in prolonged release and had a high EE%, it can be considered a promising drug delivery candidate. A study of cell proliferation on MCF10A, MCF7, and 4T1 breast cancer cells with Nio-Cur and PEG-FA@Nio-Cur3 showed, in the three cell lines, a significant decrease in the IC50 values of PEG-FA@Nio-Cur compared to Nio-Cur. PEG-FA@Nio-Cur3 exhibited higher cellular uptake efficiency in vitro compared with Nio-Cur3. MCF7 and 4T1 staining examination of the cell cycle indicated that the cells were stopped at the sub-G1 phase, and the PEG-FA@Nio-Cur induced a high apoptosis rate in breast cancer cells (MCF7 and 4T1). All in all, Cur-entrapped PEG-FA@Nio was found to be a good carrier candidate for the treatment of breast cancers.

## Data Availability

Not applicable.

## Supplementary Materials

The following supporting information can be downloaded at: https://www.mdpi.com/article/10.3390/molecules27144634/s1, Table S1. Different niosomal formulations for encapsulation of curcumin; Table S2. Primers and their sequences are used in real-time PCR; Table S3. Bax, Bcl2, and p53 PDB features; Table S4. Significant Bax, Bcl2, and p53 features in Uniport server; Table S5. Imperative features of all three genes (Bax, Bcl2 and p53 in *Homo sapiens*) from the protparam server; Table S6. Validation of three proteins (6GL8(Bcl2), 6EB6(Bax), 6SL6(p53)) by using the SAVES program; Table S7. Predicted Drug likeness of curcumin according to Lipinski’s rule; Table S8. Predicted pharmacokinetic profile of curcumin; Table S9. Molecular docking of Bax, Bcl2, and p53 genes with curcumin; Table S10. The residues in the hydrogen bond between Bax, Bcl2, p53 and curcumin; Table S11. Vesicle size, PDI, and EE % of various Cur-Nio and PEG-FA@Nio-Cur. Data are represented as mean ± SD, *n* = 3; Table S12. The release kinetic models and the parameters seen for niosomal formulations; Figure S1. Zeta potential of Nio (A), Nio-Cur (B), and PEF-FA@Nio-Cur (C); Figure S2. TEM image of the prepared optimized; Nio-Cur (A) and PEG-FA@Nio-Cur (B); Figure S3. In vitro release of Cur from Nio-Cur3 and PEG-FA@Nio-Cur3 at pH 7.4 and pH 5.4 in 25 °C; Figure S4. (A) Size stability evaluation of Nio-Cur3, (B) EE (%) stability evaluation of Nio-Cur3, (C) PDI stability evaluation of Nio-Cur3, (D) PDI stability evaluation of PEG-FA@Nio-Cur3, after two months of storage at 4 ± 2 °C and 25 ± 2 °C; Figure S5. Cytotoxicity effect analysis of MCF7 (A: vehicle (Nio), C: Cur, E: Nio-Cur, and G: and PEG-FA@Nio-Cur) and 4T1 (B: vehicle (Nio), D: Cur, F: Nio-Cur, and H: and PEG-FA@Nio-Cur) cell lines against different fabricated niosomal and non-niosomal formulation treatment in 48 h 72 h (B and E) by various concentration; Figure S6. A. Expression levels of Bax, B. Bcl2, and C. p53 genes in MCF7 cells and D. Ex-pression levels of Bax, E. Bcl2, and F. p53 genes in 4T1 cells after those breast cancer cells were exposed to Vehicle (Nio), Free Curcumin, Nio-Cur, and PEG-FA@Nio-Cur (PBGD as housekeeping gene).

## Sample Availability

Samples of the compounds are available from the authors.

## Abbreviations

Cur	curcumin
FA	folic acid
PDI	polydispersity index
EE	encapsulation efficiency
P-gp	P-glycoprotein
PPB	plasma protein binding
BBB	blood–brain barrier
VD	volume distribution
CYP	cytochrome P450
PDB	Protein Data Bank
RMSD	root mean square deviation
Rg	radius of gyration
ROS	reactive oxygen species
DCF	dichlorodihydroflourescin
