# Supplementary material for: Folate-Targeted Curcumin-Loaded Niosomes for Site-Specific Delivery in Breast Cancer Treatment: In Silico and In Vitro Study
Source: Molecules. 2022 Jul 20;27(14):4634. doi: 10.3390/molecules27144634 (PMC9322601; doi:10.3390/molecules27144634)
Supplement: Supplementary file 1 [file molecules-27-04634-s001.zip › molecules-1769089-supplementary.pdf]

molecules

Supporting Information

Folate-Targeted Curcumin-Loaded Niosomes for Site-Specific Delivery in Breast Cancer Treatment: In Silico and In Vitro Study

Banafsheh Honarvari [1,†], Sara Karimifard [2,†], Niyayesh Akhtari [3,†], Mehrnoush Mehrarya [4], Zahra Salehi Moghaddam [5], Mohammad Javed Ansari [6], Abduladheem Turki Jalil [7], Adrián Matencio [8], Francesco Trotta [8], Faten Eshrati Yeganeh [9], Bahareh Farasati Far [10], Mandana Kazem Arki [11], Mohammad Reza Naimi-Jamal [10], Hassan Noorbazargan [12], Zahra Asghari Lalami [3,*] and Mohsen Chiani [13,*]

[1] Department of Physics, Faculty of Physics and Chemistry, Alzahra University, Tehran 1993893973, Iran; banafshehhonarvari@gmail.com
[2] Central Tehran Branch, Stem Cells Research Center, Tissue Engineering and Regenerative Medicine Institute, Islamic Azad University, Tehran 15689-37813, Iran; karimifard.sara@gmail.com
[3] Department of Biology, Parand Branch, Islamic Azad University, Parand 3761396361, Iran; niyayeshakhtari@gmail.com
[4] Protein Research Center, Shahid Beheshti University, Tehran 1983969411, Iran; mehrnoush1995@yahoo.com
[5] Department of Microbial Biotechnology, School of Biology, College of Science, University of Tehran, Tehran 14155-6455, Iran; salehi.moghaddam@ut.ac.ir
[6] Department of Pharmaceutics, College of Pharmacy, Prince Sattam Bin Abdulaziz University, Al-kharj 16278, Saudi Arabia; javedpharma@yahoo.com
[7] Medical Laboratories Techniques Department, Al-Mustaqbal University College, Babylon, Hilla 51001, Iraq; abedalazeem799@gmail.com
[8] Department of Chemistry, University of Turin, Via Pietro Giuria 7, 10125 Torino, Italy; adrian.matencioduran@unito.it (A.M.); francesco.trotta@unito.it (F.T.)
[9] Science and Research Branch, Department of Chemistry, Islamic Azad University, Tehran 14515-775, Iran; ffyeganeh@gmail.com
[10] Research Laboratory of Green Organic Synthesis and Polymers, Department of Chemistry, Iran University of Science and Technology, Tehran 16846-11367, Iran; bahar.ferasati@gmail.com (B.F.F.); naimi@iust.ac.ir (M.R.N.-J.)
[11] Gastroenterology and Liver Diseases Research Center, Research Institute for Gastroenterology and Liver Diseases, Shahid Beheshti University of Medical Sciences, Tehran 19835-178, Iran; mandana.arki@gmail.com
[12] Department of Biotechnology, School of Advanced Technologies in Medicine, Shahid Beheshti University of Medical Sciences, Tehran 19857-17443, Iran; h.noorbazargan@sbmu.ac.ir
[13] New Technology Research Group, Department of Nano Biotechnology, Pasteur Institute of Iran, Tehran 1316943551, Iran
* Correspondence: zahraasghari68@yahoo.com (Z.A.L.); chiani@pasteur.ac.ir (M.C.)
† These authors contributed equally to this work.

Table S1. Different niosomal formulations for encapsulation of curcumin.

Formulation	Type of Surfactant	Lipid/Drug (mol ratio)	HLB	Transition temperature , Tc (°C)	Drug concentration(mg/ml)	Sonication time (min)	Surfactant: Cholesterol: DCP (molar ratio)
Nio-Cur1	Span20	10	8.60	16	1	5	2:1:0.05
Nio-Cur 2	Span60	10	4.70	53	1	5	2:1:0.05
Nio-Cur 3	Span80	10	4.30	-12	1	5	2:1:0.05
Nio-Cur 4	Span20	20	8.60	16	1	5	2:1:0.05
Nio-Cur 5	Span60	20	4.70	53	1	5	2:1:0.05
Nio-Cur 6	Span80	20	4.30	-12	1	5	2:1:0.05
Vehicle (Nio)	Span80	10	4.30	-12	-	5	2:1:0.05
PEG-FA@Nio-Cur 3	Span80	20	4.30	-12	1	5	2:1:0.05

Table S2. Primers and their sequences are used in real-time PCR.

Gene	Forward Primer	Reverse Primer
Bax	5’-CGGCAACTTCAACTGGGG-3’	5’-TCCAGCCCAACAGCCG-3’
BCL_2	5-’GGTGCCGGTTCAGGTACTCA-3’	5’-TTGTGGCCTTCTTTGAGTTCG-3’
p53	5’-CATCTACAAGCAGTCACAGCACAT-3’	5’-CAACCTCAGGCGGCTCATAG-3’
ß-actin	5-’TCCTCCTGAGCGCAAGTAC -3’	5’-CCTGCTTGCTGATCCACATCT-3’
PBGD	5’-ATGTCCGGTAACGGCGGC-3’	5’-CAGCATCGCTACCACAGTGTC-3’

Table S3. Bax, Bcl2, and p53 PDB features

**Target**	**Resolution**	**R-Value Free**	**R-Value Work**	**R-Value Observed**	**Total Structure Weight (kDa)**	**Atom Count**	**Mutation(s)**	**Unique protein chains**	**position**	**Gene length**
6GL8 (Bcl2)	1.40 Å	0.194	0.178	0.179	20.95 kDa	1414	yes	1	9-206	239
6EB6 (Bax)	2.02 Å	0.201	0.171	0.173	21.27 kDa	1492	yes	1	1-192	192
6SL6 (p53)	1.67 Å	0.180	0.163	0.164	28.18 kDa	1902	yes	1	89-311	393

Table S4. Significant Bax, Bcl2, and p53 features in Uniport server.

**Ligand**	**Subcellular location**		**Position of mutagenesis**		
Bax	Isoform Alpha	Mitochondrion / Cytoplasm and Cytosol	21	K → E	Reduces interaction with *Bcl2*L11, homo-oligomerization, and triggering of apoptosis
	Isoform Beta	Cytoplasm and Cytosol	74	M → D or E	Strongly reduced interaction with MCL1, *Bcl2*, *Bcl2*L1, and *Bcl2*L2.
	Isoform Gamma	Cytoplasm and Cytosol	184	S → D, E, H, or K	S → D, E, H or K: Constitutive cytoplasmic location.
	Isoform Delta	Cytoplasm and Cytosol	184	S → V	S → V: Constitutive mitochondrial location.
Bcl2	1: Nucleus membrane<br>2: Endoplasmic reticulum membrane<br>3: Mitochondrion Outer membrane		34	D → A	Abolishes cleavage by caspase-3
			64	D → A	No effect on cleavage by caspase-3.
			138-141		Loss of *Bax*-binding and anti-apoptotic activity
			144	W → A	Loss of *Bax*-binding and anti-apoptotic activity
			145	G → A	Loss of *Bax*-binding and anti-apoptotic activity
			145	G →E	Loss of *Bax*-binding and anti-apoptotic activity
			146	R → A	Loss of *Bax*-binding and anti-apoptotic activity
			188	W → A	Loss of *Bax*-binding and anti-apoptotic activity
			190	Q → L	Partial loss of *Bax*-binding and 50% decrease in anti-apoptotic activity

		191	D → A	Partial loss of *Bax*-binding and 50% decrease in anti-apoptotic activity
		192	N → A	Partial loss of *Bax*-binding and 50% decrease in anti-apoptotic activity
		194-197	Missing	Loss of *Bax*-binding and anti-apoptotic activity
		200	E → A	Partial loss of *Bax*-binding and 50% decrease in anti-apoptotic activity
p53	1: Cytoskeleton<br>2: Cytoplasm and Cytosol<br>3: Mitochondrion<br>4: Endoplasmic reticulum<br>5: Nucleus	15	S → A	Loss of interaction with PPP2R5C, PPP2CA, and PPP2R1A
		18	T → A	No effect on interaction with MDM2 and increase in protein levels after DNA damage
		20	S → A	Abolishes phosphorylation site. Abolishes increase in protein levels after DNA damage
		20	S →D	Constitutively increased T*p53* protein levels
		22-23	LW → QS	Loss of interaction with MDM2, leading to constitutively increased T*p53* protein levels
		24	K → R	Abolishes ubiquitination by MUL1
		37	S → D	Abolishes phosphorylation by MAPKAPK5
		46	S → A	Abolishes phosphorylation by DYRK2 and HIPK2 and acetylation of K-382 by CREBBP
		46	Missing	Alters interaction with WWOX
		55	T → A	Blocks phosphorylation by TAF1
		183	S → A	Strongly abolishes phosphorylation
		183	S → E	Inhibits slightly its transcriptional activity
		248	R → A	Does not induce SNAI1 degradation
		269	S → A	Abolishes phosphorylation
		269	S → E	Strongly inhibits its transcriptional activity
		284	T → E	Strongly inhibits its transcriptional activity
		291-292	KK → RR	Abolishes polyubiquitination by MKRN1
		319	K → A	Loss of nuclear localization, when associated with A-320 and A-321
		320	K → A	Loss of nuclear localization, when associated with A-319 and A-321
		321	K → A	Loss of nuclear localization, when associated with A-319 and A-320

333-337	RGRER→KGKEK	Reduced methylation by PRMT5. Reduced nuclear localization. Decreased binding to promoters of target genes. Reduced transcriptional activity
359	P → D	Abolishes binding to USP7
361	G → E	Abolishes binding to USP7
362	S → A	Abolishes binding to USP7
370	K → R	Induces a decrease in methylation by SMYD2
372	K → R	Induces a decrease in protein stabilization
373	K → R	Abolishes dimethylation by EHMT1 and EHMT2
382	K → A	Abolishes acetylation by CREBBP
382	K → R	Abolishes monomethylation by KMT5A
383	L → A	Abolishes S-315 phosphorylation by CDK2/cyclin A
385	F → A	Reduced SUMO1 conjugation
386	K → A	Abolishes SUMO1 conjugation, in vitro and in vivo
387	T → A	No effect SUMO1 conjugation
388	E → A	Abolishes SUMO1 conjugation

Table S5. Imperative features of all three genes (Bax, Bcl2 and p53 in *Homo sapiens*) from the protparam server.

gene	Theoretical pI	The estimated half-life	Instability index	Total number of negatively charged residues (Asp + Glu)	Total number of positively charged residues (Arg + Lys)
Bax	5.08	30 hours (mammalian reticulocytes, in vitro). >20 hours (yeast, in vivo). >10 hours (Escherichia coli, in vivo).	36.42 (stable)	23	20
Bcl2	6.75	30 hours (mammalian reticulocytes, in vitro). >20 hours (yeast, in vivo). >10 hours (Escherichia coli, in vivo).	51.63 (unstable)	22	21
p53	8.17	>20 hours (mammalian reticulocytes, in vitro). >20 hours (yeast, in vivo).	70.55 (unstable)	43	45

Table S6. Validation of three proteins (6GL8(Bcl2), 6EB6(Bax), 6SL6(p53)) by using the SAVES program.

	Target	ERRAT score	Verify 3D score	z-score	Ramachandran plot			
procheck					Favored region	Additional allowed	Generosity allowed	Disallowed
	6GL8(Bcl2)	100%	100%	-6.76	96.1%	3.9%	0.0%	0.0%
	6EB6(Bax)	99.4152%	98.32%	-7.99	94.9%	5.1%	0.0%	0.0%
	6SL6(p53)	88.1443%	94.12%	-6.18	93.1%	6.9%	0.0%	0.0%

Table S7. Predicted Drug likeness of curcumin according to Lipinski's rule.

Drug likeness rule	Property (unit)	Rule	Predicted Result curcumin	Predicted Result Ascorbic acid
Lipinski's rule	Molecular weight	≤500	368.38gr/mol	176.12 gr/mol
	Lipophilicity (LogP)	≤5	3.03	0.39
	Hydrogen bond acceptor	≤10	6	6
	Hydrogen bond donors	≤5	2	4

Table S8. Predicted pharmacokinetic profile of curcumin.

Category	Property (unit)	Predicted curcumin Result	ADMETLAB standards
Basic physicochemical property	LogP (partition coefficient) (log mol/L)	3.37	Favorable: 0< LogP <3<br>LogP <0: high aqueous solubility.<br>LogP >3: poor aqueous solubility.
	LogD7.4 (Distribution coefficient D) (log mol/L)	0.969	< 1: high solubility, low Permeability, and Metabolism; Permeability possible via paracellular if MW < 200.<br>1 to 3: moderate Solubility, Permeability; and low Metabolism.<br>3 to 5: low solubility; high Permeability; and moderate to high Metabolism.<br>> 5: low Solubility; high Permeability and Metabolism.
	Log S(solubility) (Log mol/L)	-4.733	Favorable: higher than -4 log mol/L<br><10 μg/mL: Low solubility;<br>10–60 μg/mL: Moderate solubility;<br>>60 μg/mL: High solubility
Absorption	Papp (Caco-2 permeability) (cm/s)	-5.052	Favorable: higher than<br>-5.15 Log unit or -4.70 or<br>-4.80
	HIA (Human Intestinal Absorption) (%)	0.569	>0.5: HIA positive<br><0.5: HIA negative
	Pgp-inhibitor	0.217	>0.5: An inhibitor<br><0.5: non-inhibitor
	Pgp-substrate	0.061	>0.5: A substrate<br><0.5: non-substrate
Distribution	PPB (Plasma protein binding) (%)	87.01	90%:<br>highly protein-bound and low therapeutic index.
	BBB (Blood brain barrier) (%)	0.814	≥0.1: BBB positive<br><0.1: BBB negative
	VD (Volume Distribution)	-0.574	Favorable: 0.04-20L/kg;<br><0.07L/kg: highly hydrophilic Confined to blood, plasma protein-bound<br>0.07-0.7L/kg: Regularly distributed;<br>>0.7L/kg: highly lipophilic; Bound to tissue components.

Metabolism	CYP1A2-Inhibitor	0.833	>0.5: An inhibitor<br><0.5: non-inhibitor
	CYP1A2-Substrate	0.504	>0.5: Substrate<br><0.5: non-substrate
	CYP3A4-Inhibitor	0.032	>0.5: An inhibitor<br><0.5: non-inhibitor
	CYP3A4-Substrate	0.406	>0.5: Substrate<br><0.5: non-substrate
	CYP2C9-inhibitor	0.071	>0.5: An inhibitor<br><0.5: non-inhibitor
	CYP2C9-substrate	-4.733	>0.5: Substrate<br><0.5: non-substrate
	CYP2C19-inhibitor	0.437	>0.5: An inhibitor<br><0.5: non-inhibitor
	CYP2C19-substrate	0.454	>0.5: Substrate<br><0.5: non-substrate
	CYP2D6-inhibitor	0.214	>0.5: An inhibitor<br><0.5: non-inhibitor
	CYP2D6-substrate	0.858	>0.5: Substrate<br><0.5: non-substrate
Excretion	Clearance (mL/min/kg)	1.541	>15 mL/min/kg: high<br>; 5mL/min/kg< Cl < 15mL/min/kg: moderate<br>; <5 mL/min/kg: low
	T1/2 (Half-life) (H)	1.687	>8h: high;<br>3h< Cl < 8h: moderate;<br><3h: low
Toxicity	hERG (hERG blockers)	0.537	>0.5: A Blocker<br><0.5: non-blocker
	H-HT (Human Hepatotoxicity)	0.796	>0.5: HHT positive<br><0.5: HHT negative

Table S9. Molecular docking of Bax, Bcl2, and p53 genes with curcumin.

	RMSD lower bond	RMSD upper bond	Binding affinity
Curcumin-Bax	0	0	-6.3
Curcumin-Bcl2	0	0	-5.0
Curcumin-p53	0	0	-6.5

Table S10. The residues in the hydrogen bond between Bax, Bcl2, p53 and curcumin.

Proteins	p53	Bax	bcl2
Residues	ARG110 – PHE 113 – LEU111 – ASN268 – GLN144 – GLN104 – HIS115 – TYR126 – ASP228 – TRP146 – PHE113 – SER90	LYS119 – LYS123 – LEU122 – LYS128 – ALA35 – THR127 – ASP84 – ILE80 – ARG34 – MET38	TYR108 – SER205 – LEU201 – ARG107 – TYR202 – ARG129 – ARG146 – ASP103 – SER205 – ARG107

Table S11. Vesicle size, PDI, and EE % of various Cur-Nio and PEG-FA@Nio-Cur. Data are represented as mean ± SD, n = 3.

**Formulation**	**Vesicle Size (nm)**	**Polydispersity index (PDI)**	**EE (%)**
Nio-Cur1	333.20±21.07	0.250±0.021	97.8778±0.2962
Nio-Cur 2	237.97±4.56	0.390±0.005	96.7111±0.3469
Nio-Cur 3	218.30±12.41	0.200±0.010	92.7111±0.4194
Nio-Cur 4	357.03±41.03	0.290±0.013	95.7667±1.3017
Nio-Cur 5	272.40±18.98	0.280±0.025	94.8222±0.0962
Nio-Cur 6	221.50±13.31	0.290±0.022	91.9944±0.0096
Vehicle (Nio)	164.80±5.42	0.180±0.013	-
PEG-FA@Nio-Cur 3	187.13±7.55	0.160±0.033	98.2517±0.7851

Table S12. The release kinetic models and the parameters seen for niosomal formulations.

Release Model	Zero-Order	Korsmeyer-Peppas		First-Order	Higuchi
	R^2	R^2	n	R^2	R^2
Free Curcumin (pH 7.4-37 °C)	0.4048	0.7030	0.3084	0.8669	0.6418
Cur-Nio (pH 7.4-37 °C)	0.8185	0.9089	0.5598	0.8771	0.9396
Cur-Nio (pH 5.4-37 °C)	0.8134	0.9326	0.4446	0.9113	0.9343
PEG-FA@Nio-Cur (pH 7.4-37 °C)	0.7915	0.9103	0.5053	0.8715	0.9209
PEG-FA@Nio-Cur (pH 5.4-37 °C)	0.7346	0.9164	0.3620	0.8764	0.8820
Free Curcumin (pH 7.4-25 °C)	0.5709	0.7732	0.5844	0.9303	0.7425
Cur-Nio (pH 7.4-25 °C)	0.8304	0.9110	0.6245	0.8720	0.9473
Cur-Nio (pH 5.4-25 °C)	0.7924	0.9128	0.4518	0.8632	0.9227
PEG-FA@Nio-Cur (pH 7.4-25 °C)	0.7689	0.9024	0.5207	0.8229	0.9059
PEG-FA@Nio-Cur (pH 5.4-25 °C)	0.7520	0.9035	0.3986	0.8590	0.8927

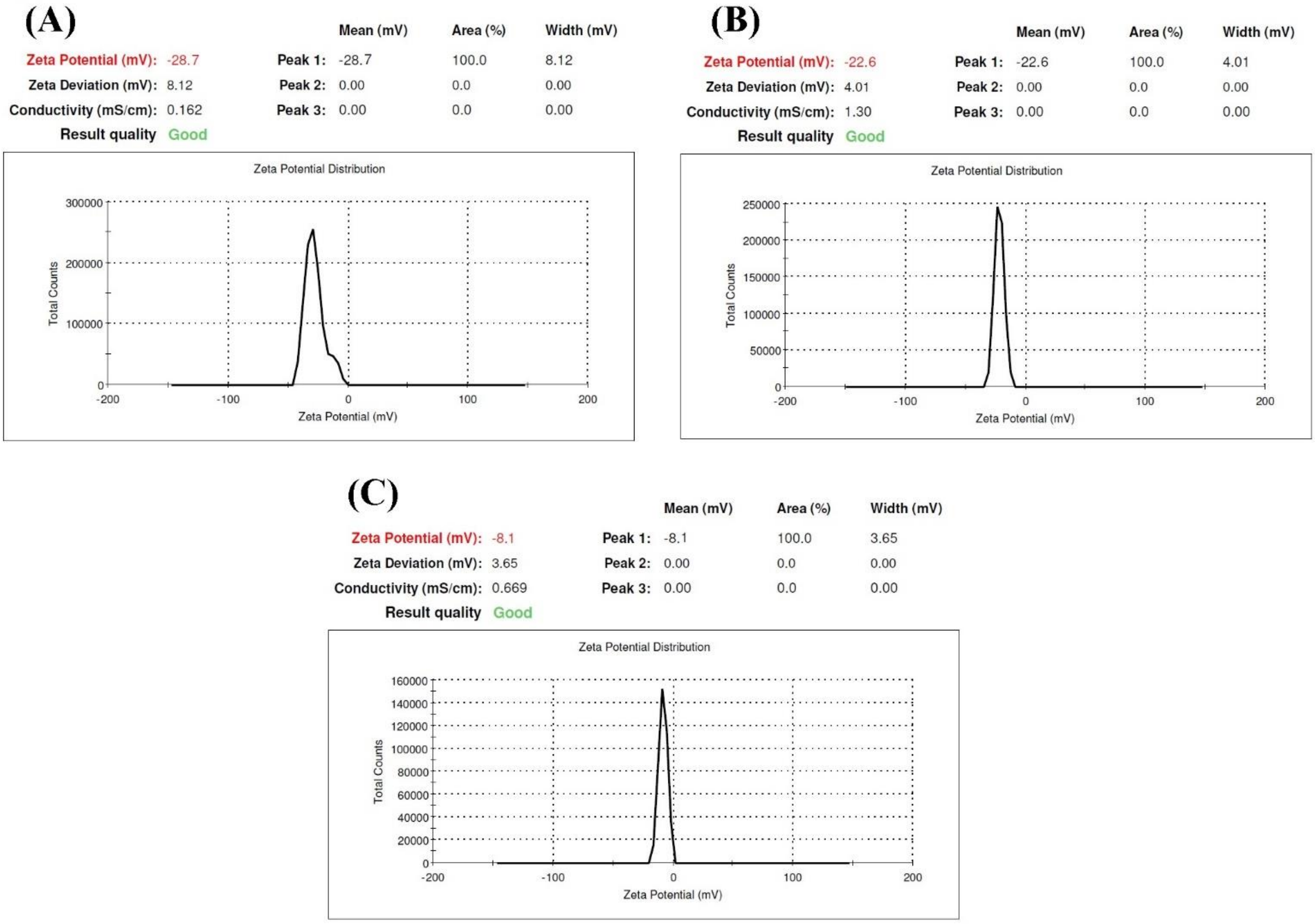

Figure S1. Zeta potential of Nio (A), Nio-Cur (B), and PEF-FA@Nio-Cur (C).

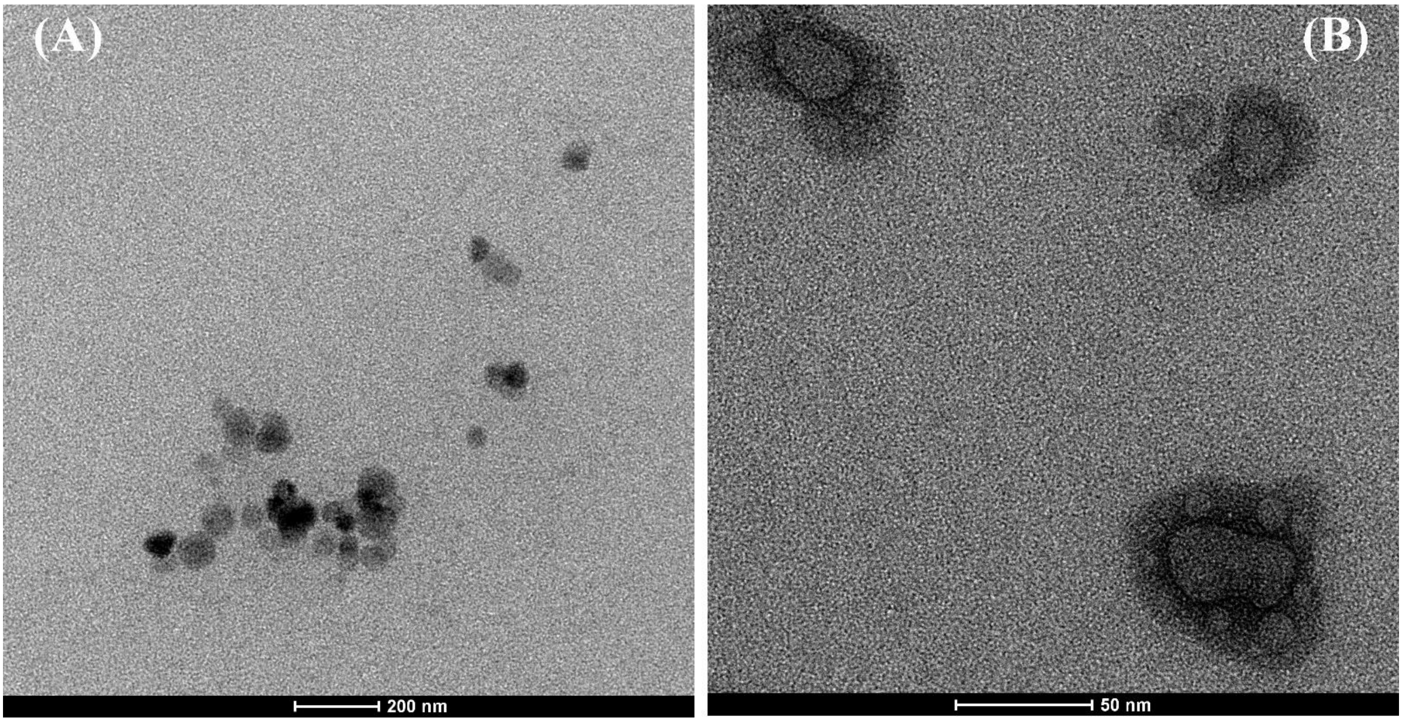

Figure S2. TEM image of the prepared optimized; Nio-Cur (A) and PEG-FA@Nio-Cur (B).

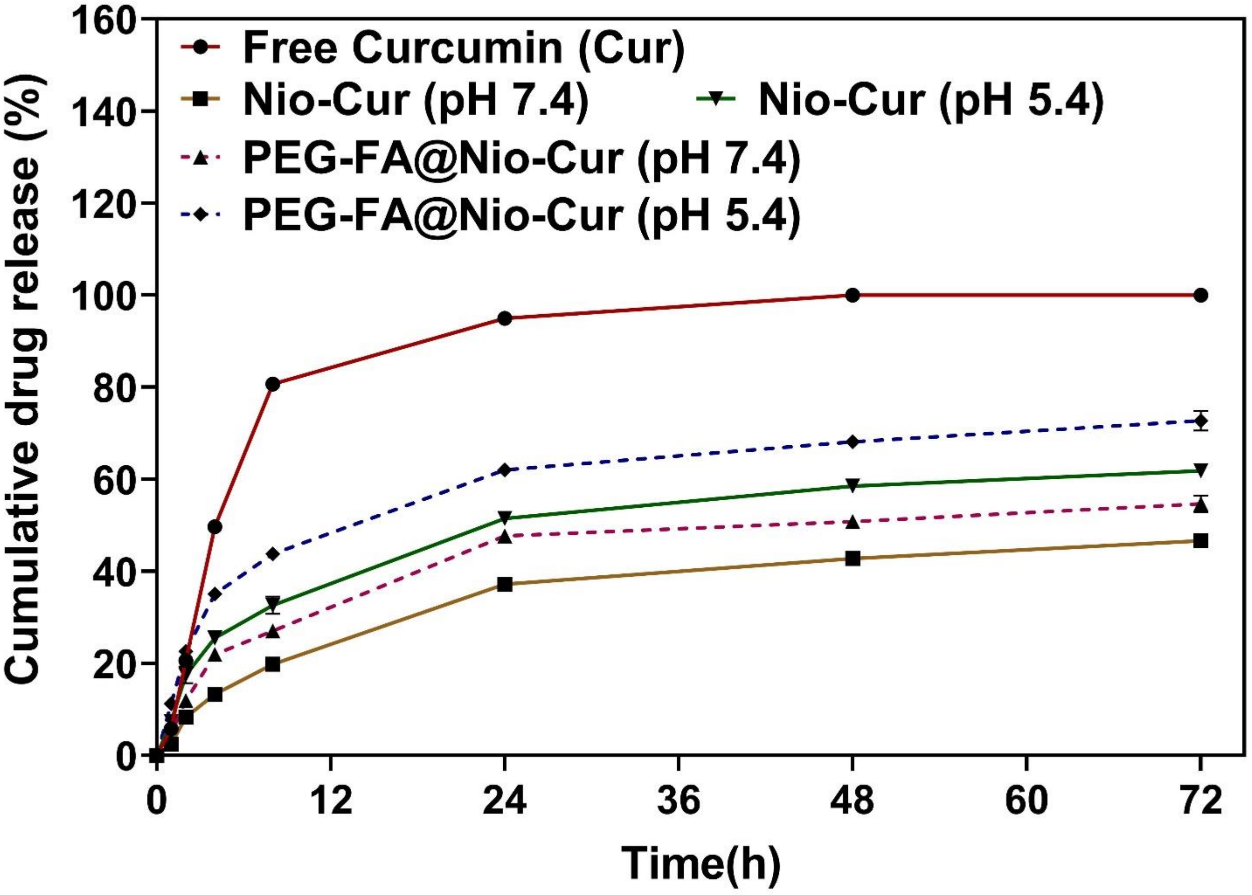

Figure S3. In vitro release of Cur from Nio-Cur3 and PEG-FA@Nio-Cur3 at pH 7.4 and pH 5.4 in 25 °C

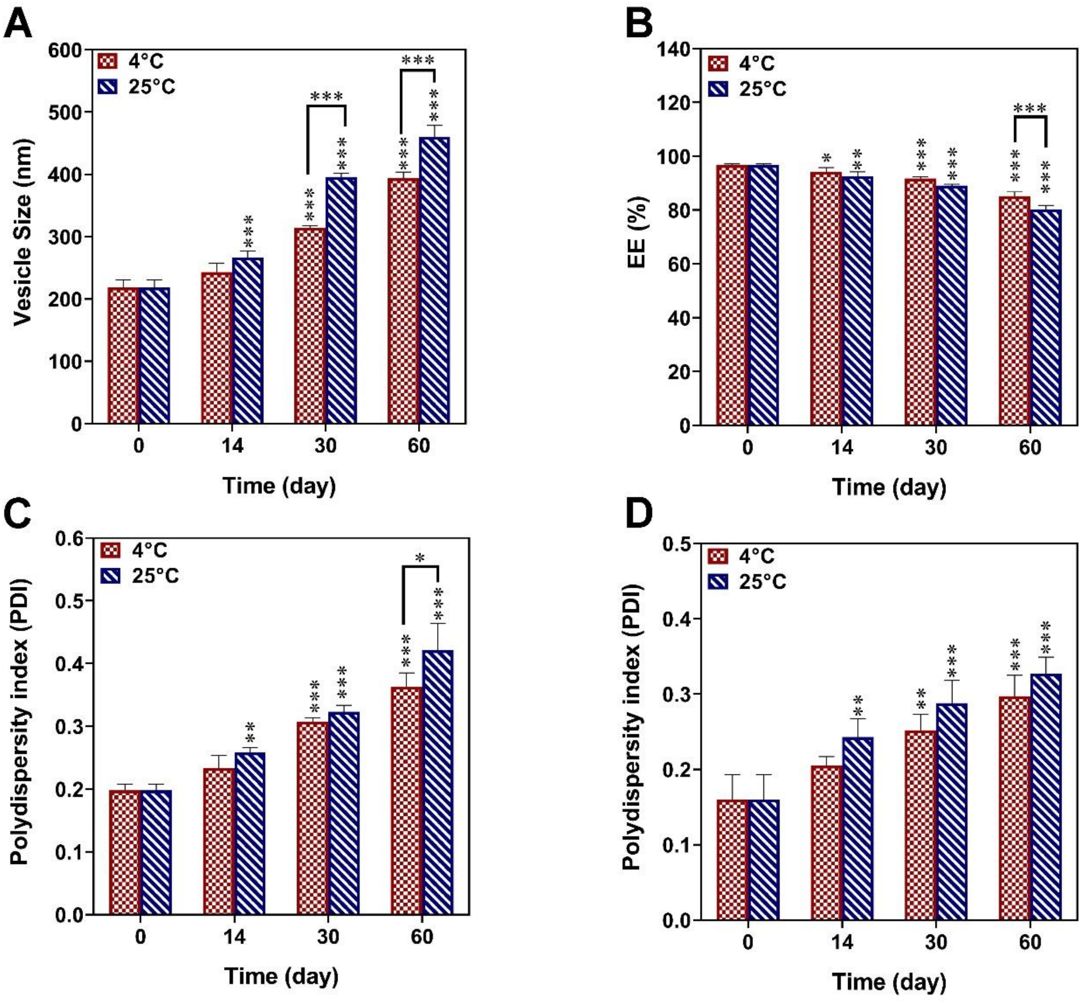

Figure S4. A) Size stability evaluation of Nio-Cur3, **B**) EE (%) stability evaluation of Nio-Cur3, **C**) PDI stability evaluation of Nio-Cur3, **D**) PDI stability evaluation of PEG-FA@Nio-Cur3, after two months of storage at 4 ± 2°C and 25 ± 2 °C. Data are represented as mean ± SD and n=3; P<.001***, P<0.01 **, P<0.05 *.

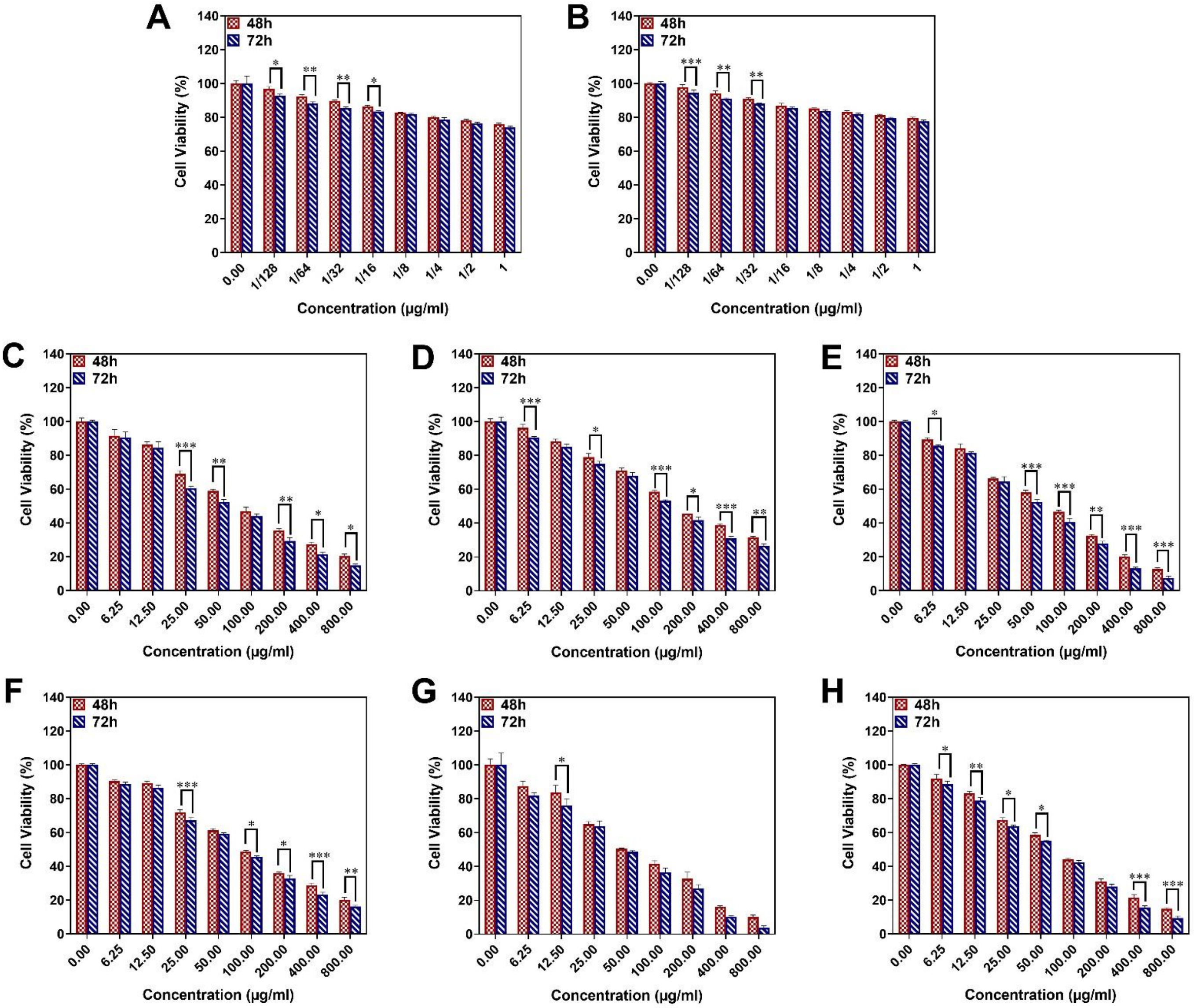

Figure S5. Cytotoxicity effect analysis of MCF7 (A: vehicle (Nio), C: Cur, E: Nio-Cur, and G: and PEG-FA@Nio-Cur) and 4T1 (B: vehicle (Nio), D: Cur, F: Nio-Cur, and H: and PEG-FA@Nio-Cur) cell lines against different fabricated niosomal and non-niosomal formulation treatment in 48hr 72hr (B and E) by various concentration.

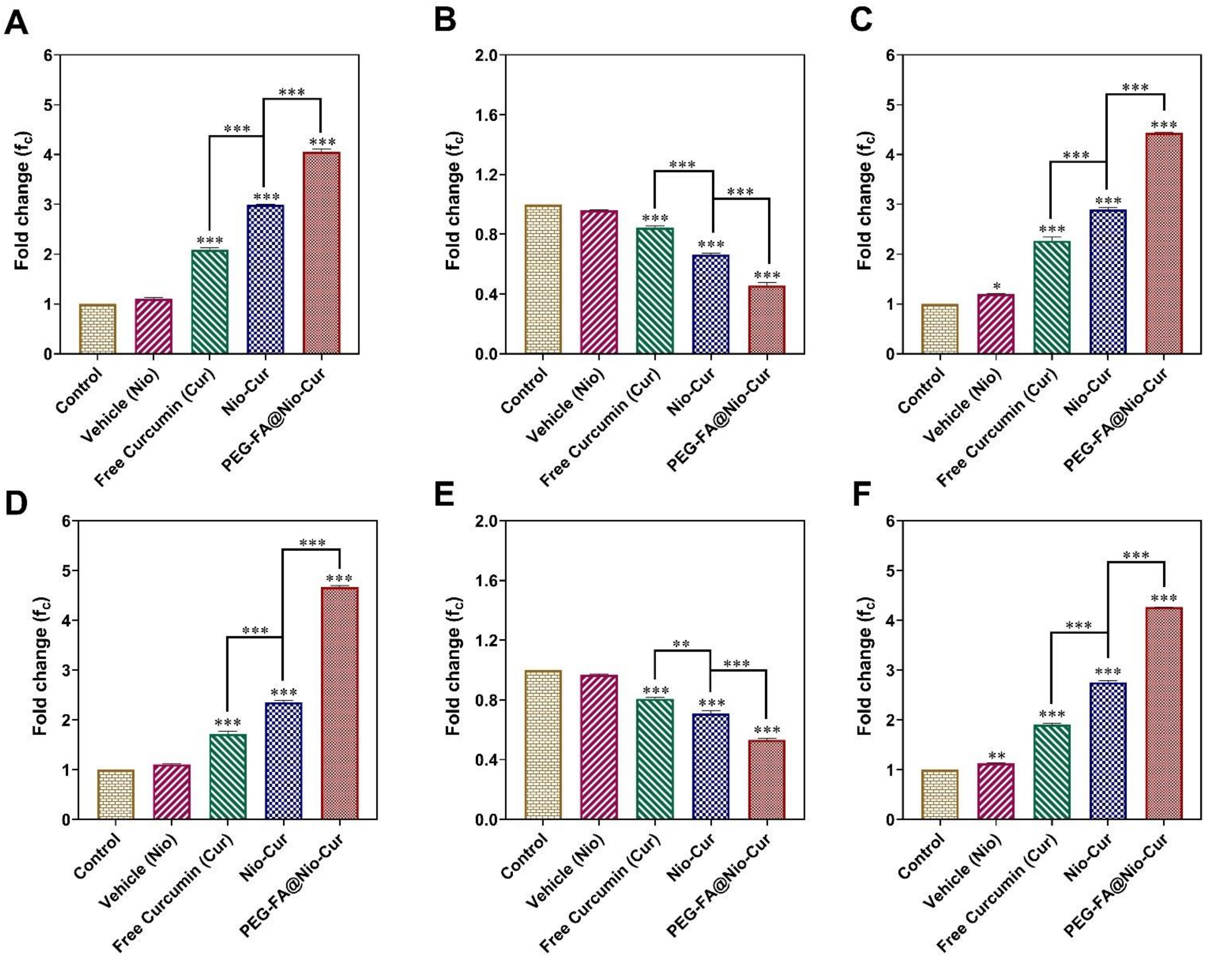

Figure S6. A. Expression levels of Bax, B. Bcl2, and C. p53 genes in MCF7 cells and D. Ex-pression levels of Bax, E. Bcl2, and F. p53 genes in 4T1 cells after those breast cancer cells were exposed to Vehicle (Nio), Free Curcumin, Nio-Cur, and PEG-FA@Nio-Cur (PBGD as housekeeping gene). Data represent means ± standard deviations (n=3). For all charts, ***: $p<0.001$; **: $p<0.01$; *: $p<0.05$.
